# Harnessing conserved signaling and metabolic pathways to enhance the maturation of functional engineered tissues

**DOI:** 10.1038/s41536-022-00246-3

**Published:** 2022-09-03

**Authors:** Neal I. Callaghan, Lauren J. Durland, Ronald G. Ireland, J. Paul Santerre, Craig A. Simmons, Locke Davenport Huyer

**Affiliations:** 1 Translational Biology and Engineering Program, Ted Rogers Centre for Heart Research, Toronto, ON M5G 1M1 Canada; 2 Institute of Biomedical Engineering, Faculty of Applied Science and Engineering, University of Toronto, Toronto, ON M5S 3G9 Canada; 3 Department of Molecular Genetics, Temerty Faculty of Medicine, University of Toronto, Toronto, ON M5S 1A8 Canada; 4 Faculty of Dentistry, University of Toronto, Toronto, ON M5G 2L3 Canada; 5 Department of Mechanical and Industrial Engineering, Faculty of Applied Science and Engineering, Toronto, ON M5S 3G8 Canada; 6 Department of Applied Oral Sciences, Faculty of Dentistry, Dalhousie University, Halifax, NS B3H 4R2 Canada; 7 School of Biomedical Engineering, Faculties of Medicine and Engineering, Dalhousie University, Halifax, NS B3H 4R2 Canada; 8 Department of Microbiology & Immunology, Faculty of Medicine, Dalhousie University, Halifax, NS B3H 4R2 Canada; 9 Present Address: Faculty of Medicine, Dalhousie University, Halifax, NS B3H 2Y9 Canada

**Keywords:** Tissue engineering, Experimental models of disease, Extracellular signalling molecules

## Abstract

The development of induced-pluripotent stem cell (iPSC)-derived cell types offers promise for basic science, drug testing, disease modeling, personalized medicine, and translatable cell therapies across many tissue types. However, in practice many iPSC-derived cells have presented as immature in physiological function, and despite efforts to recapitulate adult maturity, most have yet to meet the necessary benchmarks for the intended tissues. Here, we summarize the available state of knowledge surrounding the physiological mechanisms underlying cell maturation in several key tissues. Common signaling consolidators, as well as potential synergies between critical signaling pathways are explored. Finally, current practices in physiologically relevant tissue engineering and experimental design are critically examined, with the goal of integrating greater decision paradigms and frameworks towards achieving efficient maturation strategies, which in turn may produce higher-valued iPSC-derived tissues.

## Introduction

The scientific community is investing heavily in tissue engineering to produce highly functional models of most tissues in the human body, which both elucidate the processes underlying healthy development and function as well as the mechanisms underlying pathology. As a result, tissue engineering has achieved considerable advancements over the past decade and holds significant potential for application in cell therapy and the generation of artificial organs for implantation. Driven by a long-term vision for physiological recapitulation, a shorter-term goal for the application of engineered tissues lies in preclinical drug testing for both tissue-specific efficacy and toxicity. Another significant driver of pharmaceutical screening is the severe attrition rate of drugs in the current clinical trial paradigm. This challenge carries a significant cost burden, and is inefficient with respect to development time, animal lives, and trial participant outcomes that could be better-invested with drug candidates that were better-filtered through highly reliable preclinical models (e.g., functional human in vitro tissues as opposed to immortalized human cell lines and in vivo animal models, both of which carry certain inherent inaccuracy^1^). Moreover, post-release drug recalls demonstrate that even successfully marketed drugs may carry significant risk to the population that cannot be detected even in the medium-throughput environment of late-stage clinical trials^2,3^. By refocusing on failing drug candidates for reasons of toxicity or inefficacy earlier in the process, and passing drug candidates that may be viable in humans but not in animal models, significant patient benefit, as well as cost and time savings, could be realized in the development of dependable drugs. To reach this point, continued progress in engineering tissues that closely replicate function in vitro is required. Widespread investment in the design of organ-on-a-chip models, that allow for standardized high-throughput microphysiological system experimentation, has propelled the field toward commercial and clinical relevancy. However, the functional maturity of most engineered tissues is limited by the extent of that maturation, thereby presenting a new challenge to the field of systems modeling that does not seem to have a simple and generalizable solution.

In tissue engineering to date, special attention has been paid to tissue structure and system throughput, with both reliability and robustness recognized as essential for uptake and widespread use by both industry users and scientists^4^. Furthermore, the field has recognized the importance of comprehensive and quantitative physiological metrics by which to grade models; these are usually emergent (i.e., quantitative in themselves, but difficult to subdivide into measurable component factors that fully account for the presentation). Examples of such emergence in the form of functional physiological metrics, readouts, or endpoints depending on tissue type may include contractile kinetics and dynamics, barrier function or absorption, connectivity, electrophysiology or Ca^2+^ handling, or metabolic kinetics (biochemical output or detoxifying flux)^4,5^. These functional metrics have been ubiquitously correlated with highly specific and differentiated tissue architecture (e.g., vascularized tissues, muscle with physiologically relevant resistance, the blood-brain barrier, intestinal crypts, liver portals, etc.) as well as allowing for the recreation of relevant functional interfaces, physiological fluid flow, and selective permeability. These attempts all aim to recapitulate the tissue niche (i.e., the biochemical and mechanical local environment) as closely as possible. Furthermore, paracrine and autocrine signaling, as well as direct contact and physical signaling phenomena, may represent mechanisms underlying much of this function. Many of the traditional and high-yield aspects of tissue engineering (co-culture, 3D tissue organization) to date have been prioritized to recreate the niche. Although the physical complexity and (bio)fabrication schemes required to create some of these models has been a challenge, there have been significant advancements. As a result, recapitulating the (bio)chemical niche of those tissues is now beginning to be recognized as an active field with high-yield opportunity for iteration.

**Fig. 1:**
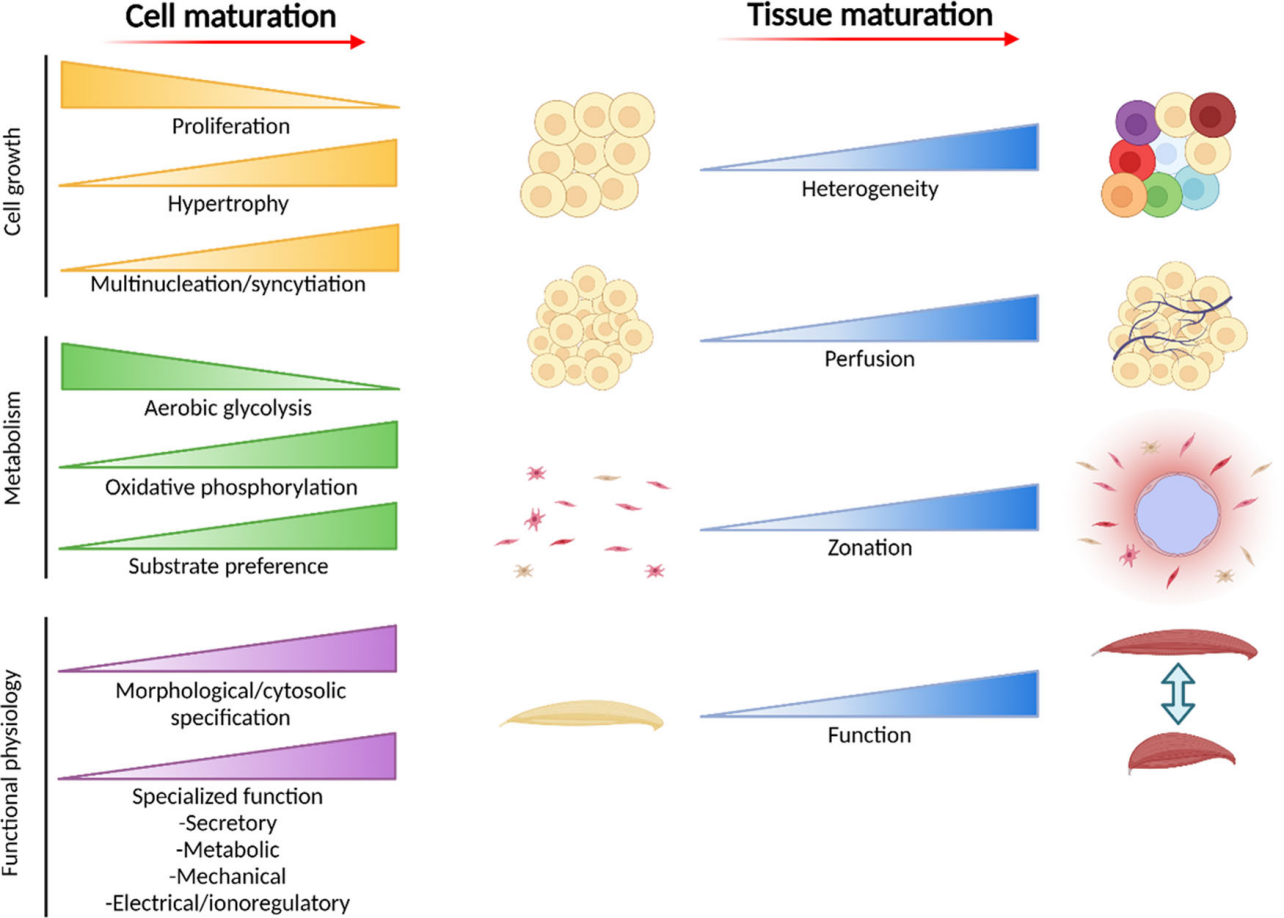
Common physiological trends in cell and tissue maturation. As metabolically provided energy is diverted from proliferative activity to physiological function, cell complexity, and functional parameters are improved. This increasing specialization necessitates an investment of energy to manage cell size, structure, and specialized structures or organelles. The energetic outlay and continued flux for this expenditure is generally maintained by high-yield and efficient oxidative phosphorylation from lipids, short-chain fatty acids, carbohydrates, amino acids, lactate, and/or ketones, depending on the specific tissue and stage of maturation in question. Metabolic supply is provided by increased perfusion and spatial zonation, at which point the tissue can then exert its hallmark function. Image created using BioRender.com.

“Cell maturation” itself is a poorly-defined process, and maturity a poorly defined state. For nearly all cells, maturity combines characteristic and interdependent states of morphology, metabolism, and cell-cycle arrest (quiescence, terminal differentiation, or senescence; depending on cell/tissue^6^) that cooperatively lend hallmark functionality to a cell for it to contribute to the physiological role of its tissue (Fig. 1). The environment conducive to cell maturation, in general, results from commonly recognized modulators of cell response and differentiation including mechanical signaling, hormone availability, and paracrine and autocrine excretions. In addition, maturing cells also benefit from metabolic substrate availability, cofactors, and other signaling-active small molecules, ionic complement, and osmotic profile, as discussed throughout the text below. As such, tissue or cell maturity in this context may best be defined as a state of signaling and metabolic homeostasis within a tissue, driven by both exogenous and endogenous stimuli, that is enabled by sufficient energetic flux to maintain the range of peak functional outputs characteristic of that tissue when enabling healthy activity, leading to functional activity.

In this review, we describe the convergence of key cell signaling pathways that regulate cell growth, extracellular matrix (ECM) deposition, terminal differentiation, and maturation of functional tissues. Given the wealth of existing in vivo knowledge associated with metabolic regulation, we refer to many excellent reviews and primary articles that offer additional detail relating to important effectors of the metabolic state. Several key recurring signaling pathways are further contextualized with insight into their potential mechanisms in evoking functional maturation. Finally, we highlight and discuss design strategies that may be well-suited to advance the function of engineered tissues. The discussion is also examined in the context of experimental design strategies developed in other engineering disciplines, in order to screen complex culture conditions using quantitative functional parameters as objective metrics. As a whole, this review defines the insight needed to yield efficient and biologically-inspired tissue engineering that could evoke maximal functional maturation without undue experimental failure or redundancy.

## Potential signaling convergences in specialized engineered tissue models: case studies

### Myocardium

The high burden of both cardiac toxicity and lack of cardiac efficacy in pharmaceutical clinical trials or clinical use, as well as the low regenerative potential of the adult myocardium post-trauma, have led to a significant investment, in both innovation and funding, toward enabling cardiac tissue engineering for screening^7–9^ and therapeutic goals^10–12^. Adult myocardium is a highly specialized tissue in structure, function, and metabolic profile, growing in complexity and metabolic profile through development and postnatal maturation^13–15^. Although many factors contributing to maturation have been identified (Table 1), their precise mechanisms and the full complement of maturation factors necessary to produce adult myocardium remain elusive. Myocardial functional specialization reflects significant changes to cell homeostasis, with extensive mitochondrial biogenesis and turnover^16^, fueled both by extreme internal energetic demands and exogenous (e.g., endocrine and paracrine) influences on cardiomyocyte (CM) structure and metabolic organization. Fully-matured myocardium retains extensive, millisecond-level regulation on mitochondrial turnover and connectivity to maintain full and efficient oxidative coverage of CM function^17^. In contrast, the diseased state, with reduced function in at least some aspect by definition, tends to necessitate a lesser degree of structural and metabolic organization and so can revert to a state resembling lower-maturity CMs in certain aspects, with corresponding decreases in function^18^. Given the significant burden of cardiovascular pathology and the shortcomings of 2D systems to recapitulate adult functional benchmarks^18–20^, 3D culture systems continue to evolve e.g.^21–24^, and often make use of concurrent electrical stimulation techniques which have been found to improve CM functionality in vitro^25–27^. As induced-pluripotent stem cell (iPSC)- or embryonic stem cell (ESC)-derived CMs can be highly matured when transplanted into adult hearts, logic would dictate that a high degree of in vitro maturation is possible (i.e., there is not a fundamental limit on the level of functional maturation attainable by PSC-CMs), although the conditions necessary to achieve it remain elusive^28^.

**Table 1:** Factors influencing myocardial maturation.

Factor	Effect on physiological maturation	Reference
Environmental		
Contact with mature myocytes	↑	In vitro^320^, in vivo^11,390^
Hypoxia	↓	^13–15,38^
Vascular perfusion	↑	In vitro^35^, in vivo^36^.
Mechanical and electrical stimulation	↑	^21,24,25,27,62^
Metabolic		
Lipid supplementation	↑	^23,38,78,79^
Glucose supplementation	↓	^81^
Galactose supplementation	↑	^53,79^
Signaling		
Triiodothyronine	↑	^38,53–57^
Neuregulin and insulin-like growth factor-1	↑	^49^
Glucocorticoids	↑	^38,55,57^
Retinoids	↑	^60^
PGC1/PPAR activation	↑	^38,39^

The myocardium is highly oxygenated, with each CM nominally directly accessible by a capillary to enable the large oxidative flux required to fuel the continuous ion transport and contractility of the myocardium. Indeed, the perinatal switch to oxidative metabolism^13–15^ seems to be mediated in part by the permanent increase in myocardial oxygenation that occurs in the switch from placental to pulmonary respiration; this rapid metabolic refocusing appears coordinated by Hand1^29^, as its overexpression diminishes fatty acid (FAO) oxidation and maintains glycolytic dependency. Hand1 is induced by HIF1α, of which perinatal downregulation during the transition to an oxygen-rich environment is well-known to be critical to the oxidative switch in the heart^30–32^. The DNA damage response to oxidative damage is a key mediator of cell-cycle arrest, and the rate of this arrest is proportional to the extent of oxidative damage^33^. Standard cell cultures, depending on cell type and density, can be chronically hypoxic or hyperoxic without obvious signs such as cell die-off^34^. Both hypoxia and hyperoxia can induce oxidative stress, as discussed below. Together, this evidence suggests that oxygen control through medium coverage^34^, as well as medium antioxidant supplementation, needs to be carefully titrated for the desired results, especially in advanced culture systems. Finally, even without perfusion, the inclusion of endothelial cells in myocardium can improve physiological metrics, both in vitro^35^ and in vivo^36^.

A key feature of perinatal CM maturation is the downregulation of proliferative processes, and the concomitant initiation of cellular hypertrophy that translates to whole-heart growth. Cardiac hypertrophy has been linked to AMPK-Sirt1 signaling, which is linked to the NAD+/NADH ratio as discussed in section 3.1 below^37^, and maturation-associated processes have been linked to PGC-1α/PPAR activity^38,39^. NAD+ availability also tends to decrease with age^40^, which therefore suggests negative modulation of cardiac differentiation and general mature phenotype as disease onset pushes CM phenotype to a neonatal-adjacent physiological state^18,41^, although actual transcriptional and proteomic states may vary. Regulation of AMPK/Akt signaling has been detected in heart tissues, including through miRNA-mediated pathways^42^. Some of this modulation may include AMPKγ isoform switching, demonstrated to mediate changes in intrinsic cardiac pacing^43^ and illustrating the importance of energetic signaling on phenotype. Important clues to homeostasis in pathology might be evident by more closely examining the metabolic phenotype. Similarly, the expression of AMPKα2 vs. α1 subunit expression rescues the heart failure phenotype, ostensibly through promoting selective mitophagy^44^. This activity may prevent energy limitation, excess ROS production, and the release of cytochrome C, the latter of which would trigger apoptosis. The authors attribute this isoform-dependent activity to the ubiquitination kinetics of the α2 vs. α1 subunits. In downstream signaling, ERRγ has been implicated in activating cardiac transcription patterns controlled by GATA, MEF2, and TEAD, while downregulating NR2C2 which itself transcriptionally represses the nuclear PPARα, retinoid receptors, and thyroid receptors^45^ that were discussed above, indicating synergies in maturation signaling. ERRs also directly activate many contractile, calcium handling, and ion channel genes indicative of maturation state^45,46^.

Several actionable biochemical effectors of CM function have been studied, and are being introduced into culture practices^20,28^. The peptide hormone neuregulin may revert to maturity and seems to increase proliferation, but also increases cardiac performance, ostensibly through ErbB signaling^47^. Additionally, the targeted inhibition of ErbB4 stops the cell cycle and induces maturation^48^. Neuregulin, a suggested myocardial regenerative agent, has also been implicated in enhancing CM maturity when used in conjunction with insulin-like growth factor-1 (IGF-1)^49^. Triiodothyronine (T3) is also known to be vital for healthy heart development^50^. Briefly, T3 increases the expression of natriuretic peptide receptors, adrenergic receptors, cytoskeletal/contractile proteins, and the complement of ion channels responsible for the development of specialized electrophysiological transients of the adult heart. T3 mediates an anabolic switch in CMs from proliferative to hypertrophic processes, likely through modulation of MAPK and Akt/mTOR pathways^51,52^, and has been widely used for physiological maturation of PSC-CMs in vitro^38,53–57^. Similarly, glucocorticoids have been used for CM maturation in vitro^38,55,57^, potentially through the activation of PGC-1α^58^. Retinoids are deeply implicated in regional development of the embryonic heart^59^, but can also promote oxidative metabolism and electrophysiological/Ca^2+^ handling maturation through unelucidated mechanisms^60^. Given the importance of retinoids in the heart field and CM subtype specification^59,61^, the continued role of retinoids in evoking higher function would not be unexpected.

Mechanical stimuli and progressive electrical pacing^21,24,25,27,62^, and Ca^2+^ homeostasis have been demonstrated to be critical in proper CM maturation. Sarcomerogenesis in particular is reliant on mechanical signaling,^63^ and sarcomeric complex-mediated mechanical signal transduction may exert significant effects on cellular disposition^64,65^. Focal adhesion/Z-disc signaling is likely another important effector of the mature CM phenotype, and often implicates Rho signaling^66–68^. In addition to its role in mediating contraction, Ca^2+^ cycling through a contractile cell may exert complex analog signaling, as described above, through PKC activation (likely tempered by lipid homeostasis and second messengers, depending on the isoforms at play). In parallel, Ca^2+^-induced calcineurin activity mediates cell-cycle arrest^69^ and in concert with the HIF activity described above may be a contributor to the switch between hyperplasia and hypertrophy in the young heart. Finally, calcineurin/LIM and cysteine-rich domains 1 (LMCD1) interactions impact cardiac development and growth^70^, but must be carefully tuned to avoid pathological hypertrophy^71^.

The heart displays a highly specialized metabolic phenotype, transitioning from a primarily-glycolytic to a high-flux and primarily-oxidative tissue over a short perinatal period^15,41,72^, as previously discussed. Healthy adult CMs demonstrate a clear preference for lipids and lactate as primary carbon sources, with flexibility toward glucose, ketone bodies, and amino acids^73^. However, the degree of flexibility and the potential interactions between substrates’ regulation of each others’ usage remains an open question, and are likely sensitive to the model (in vivo vs. in vitro, tissue vs. cell vs. isolated mitochondria) and the timescale^41,73–76^ studied. Importantly, this metabolic flexibility and overall flux seems dependent on continued Ca^2+^ homeostasis in the CM; aberrant Ca^2+^ flux via ryanodine receptor abrogation results in pathological remodeling in the cell, metabolic quiescence, and autophagy^77^. In general, there are both long- and short-term effects from signaling, remodeling, and allostery, based on substrate availability and preference with regard to cardiac function and respiration^74^. The functional effects of specific carbon sources, particularly toward maturation, are active areas of research; in general lipids seem to promote functional maturation^23,38,78,79^, but can seemingly induce lipotoxicity if not titrated appropriately^53^. From a mechanistic perspective, the removal of dietary FAs in a mouse model extended CM proliferation^80^, while PSC-CMs cultured in high vs. no glucose increased nucleotide production, maintained proliferation, and demonstrated lower maturation^81^. This evidence lends support to a ROS-induced switch from proliferation to maturation, and suggests several interesting routes of investigation to induce greater CM maturity in vitro, as well as potential targets or mechanisms to arrest or reverse pathological progression in vivo.

### Neural tissue

Although individual neural function is well-understood, the emergent properties of even small neural assemblies are very difficult to assess in vivo, with many poorly understood or even unrecognized inputs being essential to define a system. The drive to better control conditions for both applied pharmacological toxicity/efficacy screening and for basic science research into both central and peripheral nervous function and pathology have led to interest in neural tissue engineering. However, by definition, the insight garnered from a neural model is a direct function of its complexity, necessitating advanced culture techniques, and so the development of a minimum viable neural organoid is ongoing. The blood-brain barrier (BBB), formed by endothelium and glia, provides a controlled ionic and molecular environment to the neurons of the brain, ensuring proper function, despite potentially large (bio)chemical changes in the rest of the body. This relies on active transport through two layers, comprised of different types of cells (both endothelial and glial), which must transport nutrition and hormones to any neuron. This highly differentiated structure has not been ignored, and key functional metrics (e.g., excitatory and inhibitory activity) have been advanced using simple co-culture in either contact or Transwell models^82^. 3D models further improve function and give rise by definition to the potential for brain-like structure, including spontaneous tissue layer stratification within an organoid, and potentially cortical folding (gyrification)^83^, which is a morphological metric enabling greater organizational complexity. As for additional cellular-level structure, Qian et al.^83^ also review the available literature on endothelial and glial co-culture in 3D organoids. Efforts to include endothelial cells in brain organoids have resulted in gains to physiological function, spontaneous formation of in vivo-resembling developmental architecture, and the ability to engraft in vivo^84,85^. The current state is such that it is difficult to conceptualize an effective model of higher tissue functionality that does not faithfully recapitulate 3D structure and the BBB/vascular organization in doing so. Efforts to physiologically mature neural tissue are summarized in Table 2.

**Table 2:** Factors influencing neural tissue maturation.

Factor	Effect on physiological maturation	Reference
Environmental		
Co-culture with glial or endothelial cells	↑	^82^
3D culture	↑	^83–85^
Vascular perfusion	↑	^84,85^
Metabolic		
Short-chain fatty acid supplementation	↑	^93^
Ketone supplementation	↓	^93^
Signaling		
Akt activation	↑	^94–96^
AMPK activation	↑	^98^

The brain is a significant energetic sink, consuming up to 25% of total metabolized glucose in the human body^86^. Both neurons and glial cells have GLUT3 transporters^87^ that allow for direct glucose transport from the vascular endothelium. However, despite this level of energetic activity, metabolic flow and access to the brain are highly controlled by the BBB to maintain homeostasis. Neurons have high non-obligate glycolytic capacity, commensurate with their level of maturity, but their oxidative rate also appears to increase proportionally to functionality^88^, suggesting that mature function is reliant on oxidative phosphorylation. In contrast, glia are primarily-glycolytic^87,89^. Glucose can be directly imported to neurons, but due to their high relative glycolytic rate, glia surrounding blood vessels provide the generally-preferred lactate substrate via their own glycolysis^89,90^.

Astrocyte morphology is polar, with distinct subcellular compartmentalization to allow for homeostatic support of neurons. Astrocytic perisynaptic processes uptake synaptically-released glutamate using passive Na^+^/K^+^ release along their gradients; the gradient is maintained with concurrent NKA activity, and is energetically supported locally by sequestered mitochondria at these perisynaptic regions^89^. Given the low density of glial mitochondria, whole-cell activity is primarily supported by the glycolytic activity of the astrocyte which is typically elevated relative to that of the more oxidative neuron. The astrocyte then secretes waste lactate via monocarboxylate transporters, which can be used as a preferential oxidative substrate by nearby neurons (i.e., the lactate shuttle). Astrocytes also exhibit Ca^2+^ transients, ostensibly due to synaptic contact; the mechanisms underlying this physiology are still in question^91^, but these regular transients could be tied to increased metabolic demand and be, in part, responsible for regulation of the highly specific astrocyte metabolic response to a synaptic event. In a physiologically-similar process, neurons seem to exert spatial control of their mitochondria in response to intracellular glucose gradients, so as to mediate metabolic control of transport^92^. Direct signaling by metabolic substrates acting via ligands is relatively unexamined in neural tissues. However, short-chain FAs have been shown to activate GPR41/FFAR3, and by extension PLCβ/MAPK signaling, leading to the induction of sympathetic functional processes; in contrast, ketone bodies inhibit this signaling^93^, similar to in intestinal epithelium (below).

Neural development, both on a cell and tissue level, is known to be heavily mediated by the activity of the Akt pathway^94,95^ and its interactions with physical (i.e., Yap pathway) signals^96^. Akt-related signaling is also known to maintain neuronal function^97^, however, given the metabolic demands outlined above, sustained AMPK activation is also required to maintain a “healthy” neural phenotype^98^, potentially as a downstream effect of constitutive HIF2α activation^99^. It is not yet clear whether this AMPK activation is a relatively flat and continuous phenomenon, or an oscillatory pattern on an unknown timescale (see below for the role of oscillatory signals in tissue and system homeostasis). This could depend on intracellular architecture in the context of diffusion-based limitation and the need for intracellular metabolic shuttling. Nevertheless, this underscores the dipolar relationship between metabolism and the gain or maintenance of function. However, AMPK hyperactivation has been implicated in synaptic destruction via its induction of autophagic processes in cell culture and hippocampus in vivo^100^. Together, this evidence lends itself to the hypothesis that a homeostasis between AMPK- and Akt-consolidated signals provides a set of both antagonistic and synergistic controls toward coordinated maturation in neural tissue.

### Hepatic tissue

The liver, as the metabolic center of the body, carries out many functional roles both for its own maintenance, as well as whole-body homeostasis. Although well-known as the primary detoxification tissue in the body due to an array of cytochrome P450 isoforms that oxidize a wide number of small molecules, hepatic tissue also mediates glucose and lipid processing, including significant glycogen storage, FA synthesis and oxidation, and cholesterol and lipoprotein synthesis. Hepatic culture models in common use include neonatal hepatocytes, PSC-derived hepatocyte-like cells, and hepatoma cell lines (hepatocyte-like and cholangiocyte-like cells). Putative markers of hepatic tissue maturity include the formation of functional bile canaliculi featuring tight junctions, cell polarization, and the expression of drug transporter at the apical and sinusoidal poles^101–104^; advancements in evoking hepatic tissue maturity are summarized in Table 3. To date, most in vitro modeling studies have focused on the hepatocyte and increasing its functional maturation; the advancement of Kupffer cells, stellate cells, and hepatic endothelium may prove to be essential toward fully recapitulating native tissue in an engineered setting^105^.

**Table 3:** Factors influencing hepatic tissue maturation.

Factor	Effect on physiological maturation	Reference
Environmental		
3D culture	↑	^109,122,123,125^
Endothelial co-culture	↑	^101^
Metabolic		
Amino-acid enrichment	↑	^107^
Bile acid supplementation	↑	^103,110,111^
Microbially derived compound supplementation	↑	^120,121^
Signaling		
Triiodothyronine	↑	^50^
Oncostatin M and glucocorticoids	↑	^115,118^
Hepatocyte growth factor		^115–117^
Akt activation	↑	^113,114^
AMPK activation	↑	^110,111^
PKA activation	↑	^102,109,110^

The metabolic role of hepatocytes includes the esterification and circulation of absorbed lipids from diet or hepatic synthesis, as well as the partial oxidation (ketogenesis) of FAs liberated from peripheral adipocytes during low-energy periods; the latter process is substrate-regulated by negative feedback involving malonyl-CoA^106^ and is therefore limited by equilibrium except in times of extreme energetic demand (i.e., requiring whole-body ketosis). Hepatocytes cannot effectively metabolize these ketones, and so they are reserved for other tissues. Although the liver has extensive glycolytic capacity, these products are largely used for biosynthetic reactions via the pentose phosphate pathway. Instead, the liver largely subsists on oxidation of amino acids via the TCA (e.g., α-ketoacids); indeed, the provision of extra amino acids in culture medium has been shown to significantly enhance the functional output of hepatocytes in vitro^107^. The liver also produces IGF-1 in response to growth hormone; this activity is linked to hepatic maturation in vivo^108^. IGF-1 is a near-ubiquitous growth and maturation signaling agent with effects seen in many tissues as covered elsewhere in this review.

Hepatic functional maturation has been linked to several ubiquitous pathways, including PKA^102,109,110^, AMPK via LKB1^110,111^, and the MAPK cascade^111^. Physical signaling has been less-investigated in hepatic differentiation and maturation when compared to other tissues, although 3D or spatial influences have been implicated in tissue structural development^109^. Formation and maintenance of the bile canalicular network^111,112^, and its barrier function^103^, are partially regulated by bile-activated LKB1/AMPK activity. Akt has been implicated in the early phases of hepatic regeneration, in part through its role in mediating the signaling resulting from several hepatic growth factors^113^; Akt has also been tied to either developing or maintaining the metabolic functions of the liver, especially those intersecting with insulin activity and sensitivity and notably through its regulation of FoxO-controlled gene expression^114^. Many extracellular signals have been implicated in hepatic maturation. Hepatocyte growth factor (HGF) is a well-known enhancer of hepatocyte functional maturation^115,116^, whose processes are largely controlled through c-Met activity^117^. Oncostatin M acts to enhance tissue function, most likely through STAT3^115^ and in cooperation with glucocorticoid signaling, although the variance between individual glucocorticoids and their respective affinities to specific receptor isoforms makes it difficult to elucidate the underlying mechanisms^118^. Separately, T3 hormone increases gluconeogenesis and IGF excretion^50^. Although certain pathways are now well-implicated in liver maturation, it is not clear which transcription factors or direct signaling events are most closely implicated in liver tissue maturation. There is evidence that early development includes a process of gene bookmarking that can enable or enhance functional maturation^119^; atypical culture or organoid formation could therefore limit the theoretical functional ceiling of the “matured” tissue.

Many studies have suggested the importance of the local metabolic environment in liver development. The liver displays feed-forward effects of primary bile acid production, as taurocholate is implicated in maturation^110,111^. This sensitivity itself carries implications of intestinal microbiome contributions, as in intestinal cultures (discussed below). Lithocholic acid and vitamin K2 (menaquinone) compounds, which are microbially derived in vivo, induce gap junction formation^120^ and regulate CYP activity^121^. These effects in turn would seem to implicate the establishment of polarity (tying into the physical signaling discussed), as well as cell-cell communication. As suggested previously, endothelial co-culture increases maturity, including the spontaneous formation of rudimentary vasculature^101^. 3D culture improves gene expression and morphology^122^. 3D heterotypic cell-cell contact and cell-ECM interactions also induce polarization and the development of functional architecture^123^; cell spatial relations and ECM gradients also allow for metabolic zonation^124^. Direct contact and paracrine efflux from nonparenchymal cells (endothelial, stellate, Kupffer, and other immune cells) can lead to maturation in both function and physical form, or otherwise contribute to maintaining function in ex vivo primary cultures^125^.

Despite rapid advancements, disparate findings, and evolving developments in replicating hepatic structure and function in vivo, many questions remain outstanding in developing mature engineered liver in vitro^126^. These challenges include robust, replicable, and throughput-enabling cell sources for each implicated cell type (e.g., primary *vs*. PSC-derived *vs*. immortalized lines, etc.), as well as tissue architecture that would recapitulate the niche microenvironment of the target tissue. Further challenges include establishing a chemical environment conducive to gaining and maintaining tissue-level function. Although models are being established for many different hepatic pathologies using currently-available tools (e.g., hepatitis, diabetes/obesity, and cancer^127–129^), their full potential for identifying pathophysiological mechanisms and targets will not likely be realized until they can be maximally differentiated from matured, functional tissue.

### Pancreatic tissue

The ubiquity of types I and II diabetes mellitus, as well as gestational diabetes, has spurred significant scientific and financial investment into pancreatic tissue engineering, for both modeling and therapeutic applications. In developing pancreatic models, two families of function are held as primary goals: ductal or nutritional (exocrine) function, and endocrine function^130^. Towards these goals, in vitro pancreatic organoid models have been developed from both primary sources^131^ as well as PSC-derived cells^132^. Both cell sources seem able to recapitulate tissue-level structure and can demonstrate aspects of structural progression, disease modeling, and regeneration; factors implicated in pancreatic maturation are summarized in Table 4.

**Table 4:** Factors influencing pancreatic tissue maturation.

Factor	Effect on physiological maturation	Reference
Environmental		
Contact or gradient cues	↑	^133–135^
Metabolic		
Lipid supplementation	↑	^135^
Perinatal substrate switch	↑	^138,139^
Signaling		
Triiodothyronine	↑	^148^
Cortisol	↑	^147^
Pdx1 stimulation	↑	^133–135^
Akt activation	↑	^134,140^
AMPK activation	↑	^143^

As with other tissues, recapitulating native pancreatic architecture in vitro seems crucial toward achieving final mature function, and is regulated by numerous effectors, but especially the transcription factor Pdx1, which is differentially expressed via many protocols reviewed elsewhere^133–135^. Furthermore, sympathetic innervation is required for proper development and function^136^, likely due to the effects of epinephrine on regulating embryonic islet vascularization, in part by maintaining VEGF release within strict limits, which in turn mediates normal function^137^. The delivery of growth factors and ECM secretion by ECs have been suggested as key mechanisms for this activity^137^, but are likely complemented by nutrient and oxygen gradients, given the precise niche and metabolic importance of β-cells.

The pancreas undergoes a perinatal or post-weaning nutrient switch which, as with other organs, seems vital to induce higher pancreatic function^138,139^. However, the mechanisms underlying these specific adaptations or responses are not fully elucidated and are likely complex given the central signal-consolidating and influence of the pancreas in its endocrine and chemical relationships with other organs and tissues. The signaling pathways implicated in the late stages of pancreatic development and maturation seem to be held in common with other tissues; Akt/mTOR activity is implicated in post-embryonic islet secretory functional development and structure^134,140^. Furthermore, these processes are negatively correlated or reversed under high c-Myc expression, which maintains proliferative activity, and so would hypothetically oppose post-mitotic maturation processes^141^. As with many secretory cells, β-cells are relatively oxidative to provide sufficient energy flux to maintain insulinar activity. This metabolic capacity is mediated and maintained by ERRγ signaling and is thus highly implicated in islet development and sustained function^142^. Upstream of ERRγ, AMPK is implicated in both the development and functional maturation of the pancreas^143^. The Akt/AMPK balance, previously discussed, seems to hold constant in β-cells, with high mTORC1 activity often opposing metabolic specialization (e.g., the oxidative switch) and islet function (glucose regulation) in vivo^144^; the precise role of AMPK in insulinar activity remains unclear^145^, but is likely to be involved in functional maintenance, as well as pathophysiological progression, through its downstream effectors and their transcriptional regulation^146^. Several circulating hormones are likely to influence pancreatic maturation; hydrocortisone increases the expression of insulin, as well as the glucose-sensing molecules that stimulate its release^147^. Similarly to many other tissues, T3 has demonstrated an important role in embryonic and postnatal islet development and function, potentially due to its signaling effects on metabolic regulation^148^. Finally, specific GPCRs with lipid ligands may either stimulate or inhibit insulin release via PLC/PKC and adenylate cyclase pathways, including long-chain- and short-chain-specific receptors of FAs, as well as FA derivative-specific receptors with putatively insulinotropic activities, albeit through mechanisms that are not fully elucidated^135^.

Importantly, function in pancreatic cells is a product of metabolic (e.g., AMP/ATP or ADP/ATP) and Ca^2+^ oscillations in acinar^149^, α-^150^, and β-cells; Ca^2+^ transient-triggered insulinar release in the latter is well-studied as the phenomenon of “bursting”^151,152^. Common between these cell types, these Ca^2+^ transient waveforms and periodicity are highly dynamic, suggesting well-regulated consolidation of signals of various provenance to focus functional output in a dynamic and highly concerted manner. Given the importance of Ca^2+^ as a secondary messenger in many signaling processes, these transients could conceivably serve a dual purpose in directly activating maintenance of mature pancreatic structure and function. The use of linked tissue compartments on-chip^153,154^ may also prove to enhance maturation in both pancreatic and peripheral cultures, as linked tissues benefit from pancreatic secretions, while in turn the pancreas receives metabolic feedback as it seeks to establish systemic homeostasis.

### Skeletal muscle

Muscle tissue engineering efforts have advanced rapidly in recent years, constructed both to elucidate healthy function or development, as well as pathogenesis^155,156^. As surgical applications of engineered skeletal muscle tissue remain limited, the targets of most efforts are in models to better understand the development of genetic conditions such as Duchenne’s muscular dystrophy, as well as to obviate atrophy such as in ICU-acquired weakness. The contractility of skeletal muscle, as with myocardium, has spurred rapid focus on 3D culture systems and yielded many organoid designs, including innervated and vascularized models^157,158^. Key factors implicated in skeletal muscle maturation are summarized in Table 5.

**Table 5:** Factors influencing skeletal muscle tissue maturation.

Factor	Effect on physiological maturation	Reference
Environmental		
3D culture	↑	^155,156,391^
Electrical stimulation	↑	^391,392^
Nervous co-culture	↑	^157,158^
Endothelial co-culture	↑	^157,158^
Metabolic		
Glucose enrichment	↑ (fast-twitch phenotype), ↓ (slow-twitch phenotype)	^161^
Signaling		
Integrin stimulation	↑	^170–172^
Myostatin/Smad3 activation	↓	^163,169^
Akt pathway activation	↑	^163–168^
PGC1 activation	↑	^162^

Despite a seemingly similar structure to other striated muscle, myocardium, skeletal muscle presents several functional differences that necessitate important considerations for culture. Mature skeletal muscle fibers dwarf even the largest CMs, with diameters of 50–100 µm, lengths from hundreds of microns to tens of millimeters, and dozens of nuclei. Furthermore, skeletal muscle retains a high anaerobic capacity in contrast to the obligate oxidative metabolism of myocardium; skeletal muscle couples this catabolic capacity with both high gluconeogenic activity to regenerate glycogen, and a large phosphocreatine (PCr) pool in preparation for burst activity. In times of relatively low activity, skeletal muscle relies on the oxidation of glucose and lipids, using the adenylate kinase shuttle and regenerating the PCr pool for non-diffusion-limited transport^159,160^.

Mitochondrial density, and thus oxidative capacity in vivo are dependent on a trained workload. The stratification of cultured muscle to fast- or slow-twitch phenotypes has been achieved using high or low levels of glucose in culture, respectively^161^. Furthermore, ostensibly through effects on Ca^2+^-dependent signaling and/or force-feedback on maturation, streptomycin, which non-specifically inhibits Ca^2+^ channels (including force-sensitive channels), abrogates a degree of fast-twitch functionality induced by high glucose availability^161^.

The signaling effectors of engineered skeletal muscle maturation seem to resemble those of myocardium. PGC-1α upregulation increases mitochondrial density, oxidation, and contractile features^162^. PI3K, Akt, and DAG kinase activation results in hypertrophy^163–166^. The mTOR substrate P70-S6K1 is required for increased force but not hypertrophy^167^, and is a common effector of numerous steroids and growth factors (e.g., testosterone, clenbuterol, IGF-1, etc.) clinically associated with muscular maturation. Similarly, mTOR phosphorylation associated with hypertrophy and mature contractility is caused by electrical stimulation in culture^168^. Myostatin signaling opposes overdevelopment via Smad3-repression of mTOR signaling^163^; Smad3 has since been demonstrated to directly oppose PGC-1α activation and inhibit Akt through the expression of PTEN, which antagonises PI3K activity^169^. Upstream of Akt signaling, and similarly to the heart, skeletal muscle has a large mechanobiological aspect that is important toward maturation in vivo, as measured through hypertrophy and function, in part mediated by integrins and FAK signaling^170,171^. Additional mechanical signaling occurs through LMCD1 via a calcineurin-dependent pathway^172^.

It is likely that further developments in myocardial maturation and culture may benefit those domains of skeletal muscle research and vice-versa, given their similar function. However, tissue-specific energetics, mechanics, and physical form will necessitate dedicated study of both, and will likely uncover specificities in optimal culture techniques.

### Intestinal epithelium

The complexity of the gut and its ubiquity in whole-body homeostasis has inspired significant and rapidly growing research attention. Perhaps the hallmark of the gut is its largely mutualistic population of a diverse array of bacteria, which lead to a rich list of nutrients that varies with a dependence on the individual host and location along the length of the tract itself. The nutrient diversity of the intestine in vivo leads to a complex interplay between metabolism and export by the intestinal epithelium. Interest in developing new therapeutic approaches for pathologies including Crohn’s disease and irritable bowel syndrome, as well as enhancing personal nutritional benefit, have spurred considerable interest in modeling intestinal physiology in vitro. Indeed, much of our nutrition is derived from anaerobic fermentation of large molecules (e.g., dietary fiber) to short-chain fatty acids (SCFAs) by the resident microbiome^173^; recapitulating the niche of these flora will therefore likely be necessary to fully recapitulate intestinal function in vivo. To this end, there is a rich field of research ongoing in intestinal tissue engineering which has tended toward 3D organoids^174^, although both 3D and functional 2D cultures demonstrate specialized applications^175^. In the pursuit of functional intestinal synthesis, crypt differentiation has been of particular interest as of late^176^; key factors implicated in intestinal tissue maturation are summarized in Table 6. The unique niche of the intestinal lumen allowing (re)absorption and stable bacterial colonization has greatly directed the formation of unique structures. The latter lends itself to a unique definition of “maturity”; in contrast to most other tissues, “mature” intestinal epithelial cells live only for days before being shed. As a result, the crypt represents a physical gradient of maturation, with villus tips composed of highly differentiated and functional cells, while the protected crypt bottoms maintain constitutive and physiological stem or feeder (Paneth) cell co-populations allowing lifetime epithelial replenishment for intestinal function^177^. Significant in vivo differences in function exist between the small and large intestines^178^ which are out of the scope of this review given that intestinal organoid models currently lack the ability to make such distinctions.

**Table 6:** Factors influencing intestinal maturation.

Factor	Effect on physiological maturation	Reference
Environmental		
3D culture	↑	^174–176^
Hypoxia	↑	^173^
(Flora) co-culture	↑	^193–196^
Immune co-culture	↑	^197,198^
Metabolic		
Short-chain fatty acid supplementation	↑	^179,188–191^
Amino acid enrichment	↑	^178,192^
Signaling		
mTOR activation	↑	^182,184,185^
PPAR/TFAM activation	↑	^173,183^
PGC1 activation	↑	^182^

The intestinal epithelium is characterized by a dynamic environment with many gradients and biochemical triggers underlying its development. Intestinal epithelial cells sustain a high oxidative flux to metabolically support rapid proliferation and nutrient absorption. This absorption and maintenance of homeostasis coincides with a dynamic environment in the intestinal lumen, and both processes rely on active transport with high energy demands^178^. As with other tissues, the perinatal period is of considerable importance to the specialization of intestinal tissue, as the metabolic physiology of the developing intestine rapidly changes in response to diet and microbiome^179,180^, and with very specific spatial regulation^181^. Finally, the regulation of oxygen availability through PPARγ-activated beta-oxidation of lipids maintains low oxygen tension in the intestinal lumen, which helps to maintain an anaerobic environment and allows for the selection of beneficial microbiota^173^, again underscoring the importance of structure and perfusion in mediating environmental control and homeostasis in advanced tissue function. As implied above, an oxidative metabolic switch is required for full villus formation and is mediated at least in part by the mTOR/PGC1α-activated transcription factor YY1. Ablation of YY1 compromises the morphology of intestinal epithelium, as does direct pharmacological inhibition of the ETC^182^, or loss of the mitochondrial transcription factor TFAM^183^. STAT3 and mTOR signaling are inducible in intestinal cells by IL-2, and are crucial to the differentiation of several mature functional cells in organoids^184,185^.

The study of intestinal microbiota and their physiological effects has exploded recently; gut flora comprising trillions of bacteria, viruses, and fungi have been identified for far-reaching effects around the body. Most cogently, within the intestines, host-microbe and microbe-microbe interactions alter gut metabolism, absorption, and immunity, which can promote homeostasis or diverge toward disease^186,187^. A key nutritional role of the intestinal microbiota is in the partial fermentation of primary nutrients to produce SCFAs (chiefly acetate, propionate, and butyrate), which are more readily uptaken by the intestinal epithelium^188^ and form a non-trivial source of energy for the body, in addition to the monosaccharides, FAs, cholesterol, and amino acids more commonly attributed to nutrition. These SCFAs exert immediate and notable effects on the local inflammasome and epithelial physiology; dietary fibers processed by the microbiome into SCFAs increases intestinal intracellular [Ca^2+^], mediates epithelial repair through IL-18, stabilizes HIF1, and induces STAT activity^189^.

Microbial SCFAs also activate GPR109A/HCAR2 in gut epithelium and, similarly to GPR43/FFAR3, this receptor carries complex and possibly tissue-dependent interactions with ketones^190^. GPR109A activation influences the inflammasome, ion homeostasis, and gut wall integrity and barrier function; this interaction may be critical in the development of colitis/fiber nutrition models^179,188–190^. Furthermore, GPR43 stimulation by SCFAs may also promote antimicrobial peptide production in IECs via mTOR and STAT3 signaling^191^, which again seem to represent ubiquitous effectors of maturation.

The amino acid l-glutamate is a key nutrient in intestinal metabolism from birth onwards. There is a strong negative l-glutamate gradient from the gut to the bloodstream, due to local oxidation and consumption as a precursor for the biosynthesis of many amino acids; unabsorbed glutamate from the small intestinal lumen can become a precursor for additional SCFA production in the colon^178^. Perhaps unsurprisingly, evidence is also accruing that other amino acids carry out key physiological signaling roles as well^192^, suggesting that as tissue-engineered models further evolve, maintaining a range of available metabolites will be key to evoking and detecting precise functionalities.

Due to the essential and ubiquitous role of the microbiome in intestinal function, intestinal/floral co-culture is a rapidly growing field and at the forefront of establishing steady-state colonization models of culture. Models associated with the study of the leading risk factor of fatal gastrointestinal disease, *H. pylori*, have been developed, along with other commensal or pathogenic bacteria (e.g., Clostridium, *Salmonella*, etc.), enteric viruses, and different stromal co-cultures to encapsulate intestine functionality in a larger context^193–196^. Additional efforts undertaken with immune co-culture within intestinal organoids^197,198^ suggest great promise, both at dissecting healthy function as well as modeling autoimmune or pathogen-linked disease.

### Kidney

Nephrotoxicity remains a significant issue underlying clinical trial failures and drug recalls, as well as a persistent risk, even in marketed drugs; the need for relevant preclinical screening infrastructure and personalized risk profiling for prospective drug regimens has spurred significant effort in developing iPSC models of renal function. The shortage of transplantable kidneys has also spurred significant effort in lab-grown, artificial, or wearable tissue-engineered kidneys. Current engineered kidney organoids appear to recapitulate much of the cellular heterogeneity and basic architecture of the developing kidney^199^. Moreover, many of these differentiation and spontaneous structuring processes seem to mirror in vivo developmental processes based on molecular identities and gradients; such identified factors are summarized in Table 7. Despite these similarities, organoids do not approach mature function, and may in fact regress or fibrose before gaining such advanced filtering and barrier functions^199^.

**Table 7:** Factors influencing kidney tissue maturation.

Factor	Effect on physiological maturation	Reference
Environmental		
3D culture	↑	^199^
Endothelial co-culture	↑	^203^
Metabolic		
Glucose supplementation	↑ or ↓ based on region	^202^
Signaling		
ERBF-1	↑	^205^.
ERR activation	↑	^204^
Akt activation	↑	^200^
AMPK activation	↑	^200^
PPAR activation	↑	^201^

As in other highly differentiated tissues, the structures of the postnatal kidney in vivo are known to be highly oxidative. In fact, the kidney is efficient enough to be a net positive source of glucose during energy restriction (i.e., highly-developed gluconeogenic capacity supported by oxidative phosphorylation). However, there are established heterogeneity of metabolic activity and substrate preference along the nephron, wherein proximal tubules are the most oxidative, given the ongoing need for active transport for resorptive processes; the development of these spatial energetic specialization domains has been linked to Akt/mTOR and AMPK signaling^200^, and is seemingly maintained by differential PPAR expression^201^. There is additional capacity for glycolytic futile cycling, potentially for glucose or temperature regulation, or more likely to provide a nutritive buffer for the constantly changing demands on the kidney due to a number of activities (e.g., exertion, eating, drinking, excretion, etc.) that manifest in fluctuating electrolyte levels^202^.

The signaling effectors underlying the provenance of these spatially distinct functional regions in the nephron remains unclear. Multiple gradients (e.g., chemical, osmolar, nutritive, and oxygen availability) exist and largely correlate in the nephron, which could therefore benefit from many of the signaling paradigms discussed elsewhere in this review, to aid in maturation and stratification. Furthermore, as iPSC-derived renal organoids can spontaneously self-organize into nephron-like structures with little functional pressure and even in the absence of perfusion^199^, there is likely a large physical or spatial signaling component to early nephric tissue maturation. This spontaneous architecture can be complemented with vasculature that is perfusable after implantation^203^. Other potential transcription factors at play carry traditionally significant metabolic effects; ERRγ has been demonstrated in renal epithelial cells to mediate mitochondrial development, fueling collecting duct function and resorptive processes (e.g., glucose, amino acids, and electrolytes) in cooperation with hepatic nuclear factor-1β^204^. Furthermore, early B-cell factor-1 expression is spatially-organized, mediating glomerular structure, podocyte differentiation, and metabolism^205^. The induction of these effectors is still poorly understood and will likely benefit from the use of new organoid culture systems in concert with traditional loss-of-function studies and high-throughput screens, so as to rapidly identify the key stimuli that seemingly induce and modulate kidney maturation.

## Common central signaling trends underlying functional maturation

**Fig. 2:**
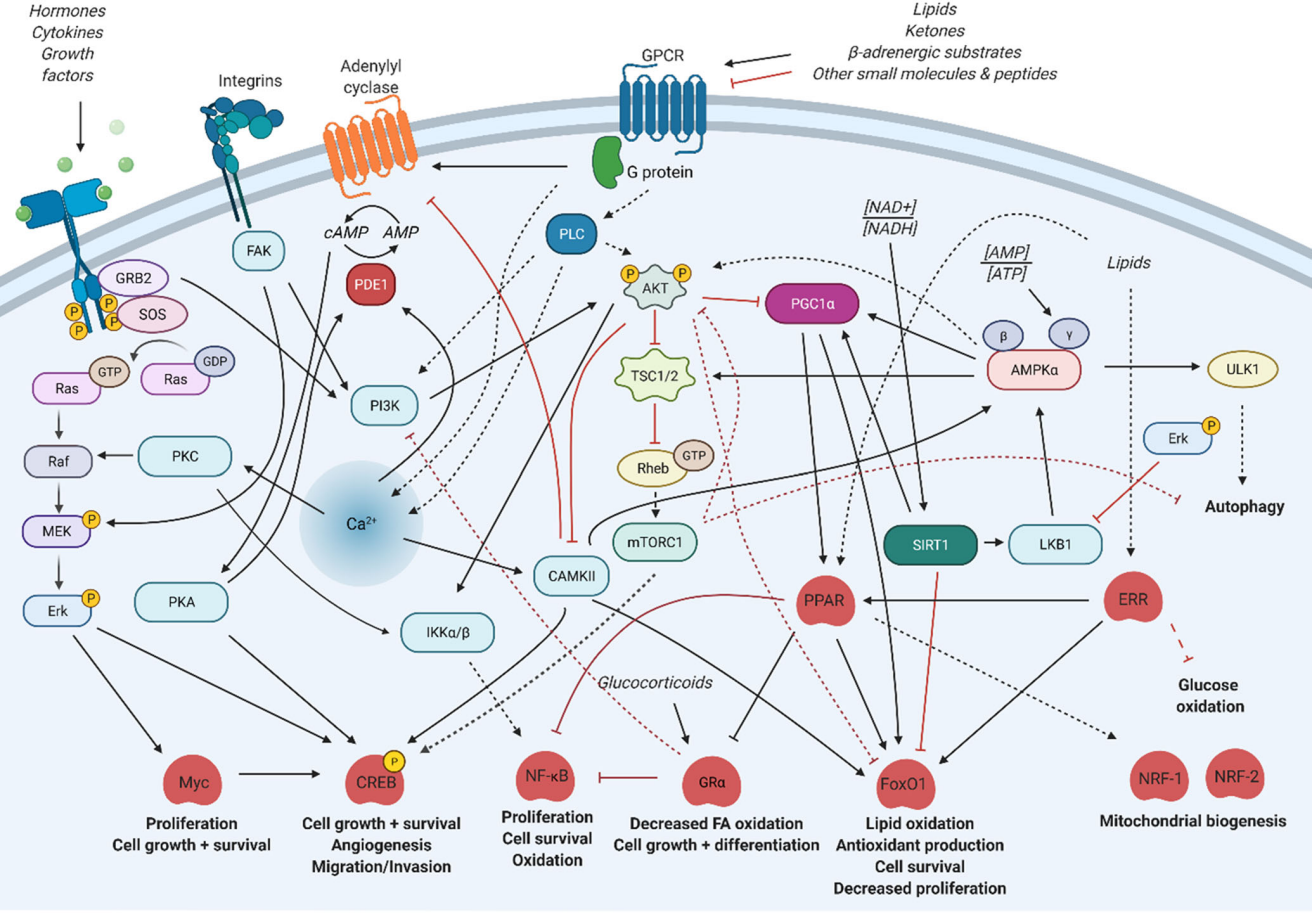
Simplified visualization of common convergences between ubiquitous signaling pathways influencing tissue maturation. Black arrows represent activation while red hashes represent inhibition; solid lines represent putatively direct interactions while dashed lines represent indirect effects (intermediates not shown or unknown). Image created using BioRender.com.

Much of the maturation and developmental progress of tissues have been attributed to Akt and its effectors (often investigated via mTORC1, its subunit Raptor, and its substrates 4E-BP1 and p70S6K) through their enhancement of differentiation, motility, and proliferative cellular processes. This activation has generally been found to be conserved across tissue types, driving direct signaling cascades to regulate cell function through existing infrastructure. In the context of maximizing tissue maturation and functional performance, this supports the existing experimental evidence paradigm that a somatic cell source could be critical to the efficiency and ceiling associated with functional performance in iPSC-derived cells^206,207^. However, certain roles for Akt (also known as protein kinase B) would seem to preclude it as the sole major driver of maturation, especially as it pertains to metabolic maturation (typically increasing catabolic flux capacity and activity to fuel matured functional energetic demands). This suggests AMP-activated protein kinase (AMPK) as a key player in maturation, due to its role in mitochondrial biogenesis and inducing catabolic pathways to balance and tune the primarily anabolic and anti-quiescent role of Akt (Fig. 2). Throughout this review, common and specific effectors of cell or tissue maturation will be identified, suggesting that the active epigenome and larger patterns of expression are key to specifying the important tissue-specific processes that occur during maturation.

### Consolidators of metabolic signaling: the Akt and AMPK axes

Akt is a central signaling consolidator linking upstream insulin signaling and phosphoinositide signaling (receptor tyrosine kinases, G-protein coupled receptors, focal adhesion kinases, Janus kinases, B-cell receptors, etc.) to control of mTOR, GSK3, MAPK, IKK, nitric oxide, and Bcl signaling, among others; its activation canonically results in cell survival and DNA repair, growth and anabolic metabolism, motility, and proliferation from this wide array of regulators and effectors^208^ implicated in cell growth, proliferation, differentiation, and alternatively apoptosis. Akt is subject to positive feedback from mTORC2 but potentially negative feedback from mTORC1^114,209,210^.

In contrast, the heterotrimeric serine/threonine kinase complex forming AMPK is generally perceived to induce widespread catabolic processes that serve to increase intracellular substrate availability and flux. Its catalytic activity is mainly controlled by phosphorylation of Thr172 on the activation loop motif of its α-subunit by liver kinase 1 (LKB1)^211^ and calcium/calmodulin-dependent protein Kinase (CaMKK)^212^. The kinetics of this phosphorylation are positively influenced by the binding of AMP to the Bateman domain of the AMPK γ-subunit, which itself is controlled by the AMP/ATP ratio of the cell. This ratio is an inverse indicator of the cells’ energy state. LKB1 itself is activated by PKA (adenylate cyclase produces cAMP), as well as Erk and p90RSK from the MAPK cascade^213^. CaMKK, in contrast, is activated by increased intracellular [Ca^2+^] resulting as a second messenger from a number of cell signals. AMPK is also directly inhibited by Akt phosphorylation on the serine-threonine-rich loop at Ser487, but only on AMPKα1 (i.e., the α2 isoform cannot be negatively regulated by Akt)^214^. This isoform dependency lends itself to state-dependent and tissue-specific mechanisms of controlling crosstalk between pathways. In turn, AMPK both activates TSC1/2 and inhibits Raptor, thus preventing both the propagation of Akt signaling as well as the positive feedback loop of Akt activation through mTORC1/2 cycling^215–218^. Unregulated AMPK activation causing continued catabolism leads to autophagy through targets including activation of ULK and inhibition of p70S6K.

By upregulating catabolism, AMPK indirectly regenerates NAD+ by enhancing NADH consumption via the electron transport chain (ETC), which in turn activates the NAD+-dependent deacetylase SIRT1. Important targets of both AMPK and SIRT1 are PPARγ coactivator-1α (PGC-1α), forkhead box O (FOXO), eukaryotic initiation factor-2 (eIF2) and peroxisome proliferator-activated receptor-γ (PPARγ)^211,219^), which are also control points of phosphoinositide/Akt signaling. Furthermore, AMPK directly activates catabolic pathways (e.g., glucose internalization and glycolysis, FAO), and inhibits anabolic pathways by phosphorylating key control point enzymes^220^.

AMPK exerts additional significant transcriptional control through the PPARγ-coactivator-1α (PGC1α) signaling cascade, directly or indirectly activating transcription factors including peroxisome proliferator-activated receptors PPARα and PPARβ, nuclear respiratory factors (NRF), and estrogen-related receptors (ERR; see discussion below)^16,45^; these gene products enhance mitochondrial biogenesis, lipid uptake and beta-oxidation, ketogenesis, gluconeogenesis, and bile production^41,73^. FOXOs, phosphorylated through AMPK/PGC1α, also upregulate gene products to protect against oxidative stress, both in short-term response to direct oxidative upregulation, as well as the increased oxidative baseline brought on by AMPK-induced transcriptional programs and mitochondrial biogenesis^220^.

As mentioned previously, SIRT1 is a vital indirect effector of AMPK signaling via catabolic regeneration of its allosteric activator NAD+. SIRT1 increases mitochondrial function via PGC-1α deacetylation, acting through both NRF1/2- mediated mitochondrial biogenesis and CAC/ETC gene upregulation^211,221^, as well as increasing insulin sensitivity^222^. SIRT1 is kinetically limited by NAD+ availability^223,224^, which is dependent on both the energetic and redox states of the cell; stresses associated with both increased SIRT1 activity due to NAD+availability as well as activation of the AMPK/PGC/PPAR axis. PGC1α also enhances MCT expression^225^, consistent with its role in enhancing catabolic activities. SIRT1 furthermore displays feed-forward expression regulation where it upregulates activity of its own transcriptional activators^224^. Similarly, SIRT1 deacetylates LKB1, causing nuclear-cytoplasm translocation and allowing it to be directly bound and activated by MO25 and STRAD^226^, whereupon it can further enhance AMPK activation.

The adiponectin receptor 1 (AdipoR1) is another common means by which cells serve to activate catabolism; AdipoR1 activation increases intracellular [Ca2^+^], thereby activating CaMKKβ^227^ which is an effector of the activating phosphorylation event of Thr172 of AMPKα^228^; AMPK would therefore activate PPARα activity through the PGC1α/SIRT1 axis outlined previously. AdipoR2 in contrast appears to more directly activate PPARα, potentially through increasing its ligand availability, although the precise mechanisms of this action remain unclear^227^.

While Akt and AMPK are usually portrayed as mutually opposing signalers, with common targets that often provide negative feedback for each other^229^, there are conceivable instances in which these processes can induce each other^230^, whether directly or indirectly. For example, under very specific circumstances with exogenous hormones combined with metabolic/hypoxic stress, AMPK initiates Akt activity by inducing ubiquitination^231^. Perhaps more interestingly, despite their common opposition, both Akt and AMPK activities support glucose uptake and mitochondrial biogenesis^232–235^. This is in keeping with their synthetic/anabolic and energy-mobilizing roles, *respectively*, although direct convergence of these pathways has yet to be thoroughly characterized in the production of robust and efficient mitochondria. Similarly, the complex interplay between Akt and AMPK suggests that the highly regulated physical, biochemical, and functional niches of many developing tissues could in theory benefit from closely regulated crosstalk between these two master regulators of cell function. The convergence of this signaling represents a key control point in the functional maturation of tissues, and one that can be influenced by highly regulated temporal signaling to tune physiology in real time. In the next section, we discuss the cellular digitization of analog signaling, and the role of oscillating signals in the putative balancing of opposing AMPK and Akt signaling processes. The latter are called on to precisely regulate cell maturation and function.

### Oscillating signaling in metabolic homeostasis and cell maturation

The absolute ATP content within a cell is low^236^, meaning that it must be recycled rapidly. Contracting cells, such as muscle, only contain ATP sufficient for a few contractions^237^, while cells involved in transport, homeostasis, or metabolism for tissue function (e.g., neurons, intestinal epithelium, glomerular cells, pancreatic β-cells, hepatocytes, activated immune cells, etc.) must match a high continuous metabolic drain, generally only sustainable through efficient energetic organization (i.e., oxidative phosphorylation). Such energetic infrastructure would include high capacity for O_2_ transport to the cell, high/O_2_-sequestering capacity, high oxidative substrate uptake, high mitochondrial content and substrate import capacity, and direct adenine nucleotide channeling (DANC) or PCr systems to rapidly disperse chemical energy throughout the cell^13,238^. Cyclic ATP shortages will activate AMPK on a similar oscillating basis, inducing increased [NAD+] and activating SIRT1. Importantly, [NAD+] is de facto tied to the redox state of the cell; changes to redox homeostasis within the cell will necessarily impact AMPK activation. Additionally, there is mounting evidence that oscillations of ERK activation (frequency and amplitude) significantly affect transduction into its physiological effects^239–242^.

A key second messenger in many signaling pathways is Ca^2+^, with direct roles in PI3K/Akt/NFAT pathways, as well as those of certain PKC isoforms. Ca^2+^ oscillations have been detected in many cell types^151,152,243,244^, which when compared to modular but flat levels offer notable benefits to these ubiquitous signaling pathways. The functional effect of Ca^2+^ oscillations is that a binary signal (i.e., [Ca^2+^]_i_ above or below a threshold for enzyme activation) is evolved into a digital signal modulated by many control points^245^. Moreover, PKC activity can be sustained well after the dissipation of an initial activating Ca^2+^ transient due to dynamics of isoform control, diacylglycerol (DAG) availability, and cytosolic trafficking^246,247^, which increases the information density of a PKC signaling event and which has been studied mostly in the context of neural long-term potentiation. The result of discrete Ca^2+^ events is very fine control over Ca^2+^-sensitive transcription or other signaling processes^244^, with the opportunity for further signaling complexity offered by crosstalk with other signals as discussed above. Modeling efforts have demonstrated that oscillating Ca^2+^ in the form of cytoplasmic transients will result in very different active patterns of dephosphorylated, DNA-ready NFAT, which will interact differently with second messenger-originating Ca^2+^ (e.g., from hypertrophic signaling pathways)^243^. For example, cAMP oscillations can provide filtering to fine-tune oscillating Ca^2+^ signals^248^ https://journals.plos.org/plosone/article?id=10.1371/journal.pone.0007189. In the case of endothelium, pulsatile flow in arterial vascular constructs (already known to be associated with endothelial proliferation, function, and patency^249,250^) induces Ca^2+^ oscillations of much greater magnitude than steady shear stress^251^, leading to extensive Ca^2+^ transient models in endothelial cells (ECs)^252^ that provide valuable insight into the incorporation of endothelial structures into co-cultured tissue models. Shear forces are well-known to be important in endothelial monolayer proliferation, function, and patency. Furthermore, these Ca^2+^ oscillations are dependent on VEGF signaling correlated to angiogenesis^253^. Finally, as Ca^2+^ channels are well-known to operate with higher-order kinetics with respect to [Ca^2+^]^254–256^, the ionic environment both within and outside of the cell becomes clear as a dynamic modulator of physiological signaling.

Therefore, interactions of cyclic metabolic signalers could be similarly expected to exert highly specific controls in concert with exogenous growth or maturation signals. For example, increased cytosolic [Ca^2+^] upstream of the AMPK activator CaMKK should cyclically induce catabolism to fuel contractions in muscle cells, while PKA interacts with AMPK through adenylate kinase^257^. Similarly, there could conceivably be Ca^2+^-facilitated PI3K or PKC signaling conflicting with AMPK activation. Considering a muscle cell as an example, PI3K activation can be expected to occur cyclically through integrin/FAK signaling during contractions. These seemingly contrasting signals are likely highly tuned and sensitive in vivo to provide maximum metabolic control, giving further impetus to mirror the (bio)chemical, ionic, and mechanical niches of engineered tissue models to maximize their transferability.

There are obvious difficulties in trying to assess cyclic activation of AMPK, Akt, or other signaling consolidators. Nevertheless, new work has demonstrated a rapid induction of AMPK activation (<30 s) and signaling in newly-activated neurons^98^, with corresponding long-term effects that presuppose continual AMPK activity. Although many effects of AMPK are direct activations and inhibitions of existing transport and catalytic processes, it is prudent to think that short but periodic upregulations of specific genes will in time change the proteome of the cell appreciably. For example, it could be conceived that short activations of AMPK, above a certain analog threshold, result in a low near-constant digital signal. This signal could manifest in certain long-term AMPK effects resulting from a convergence with Akt activation patterns (e.g., robust mitochondrial biogenesis), while avoiding certain counterproductive AMPK-linked activities in an otherwise maturing or differentiating cell (e.g., low-integrity (leaky or inefficient) mitochondrial biogenesis caused by selective gene expression, or autophagy and excessive nonoxidative catabolism). Oscillations or aggregate effects of cyclic metabolic demand such as exercise could conceivably exert differential effects on AMPK activation patterns^258^. Similarly, insulin control in the body is a quintessential example of oscillating endocrine signaling, with pancreatic secretions affecting whole-body metabolic homeostasis and carbon sourcing^152,259,260^; prandial rhythms or nutritional status variation in other hormone profiles would provide additional control points from the same base stimulus (e.g., feeding).

Perhaps one of the most stable and pervasive oscillating signals in vivo is circadian rhythms, which are well-established effectors of maturation in longer timescales. These signals control a wide range of internal cell processes, and greatly influence the secretory processes of different cells, thereby modulating the levels of different hormones and other circulating factors throughout the day, with downstream effects on cellular metabolite and Ca^2+^ concentrations^261^. Circadian rhythms and their signals have been implicated in both myocardial^262^ and β-cell maturation in vivo^263^. Although little in vitro work has examined the effect due to notable challenges of both controlling and modulating the cellular clock in culture, co-culture models may be able to recapitulate some degree of these interactions, possibly potentiating the impact of advanced culture techniques toward greater maturation.

### Physical signaling and cell-cell contact in maturation

The physical environment of cells in their functional development, plasticity, and response to stimuli has been of considerable interest, as differential mechanics are well-known to be both the source and result of a number of pathologies across many tissue types^264–268^. Cell-mediated physical forces have been extensively identified as critical to cell fate and development from the early embryonic stage^269,270^, wherein several examples of pathology can be attributed to aberrant processes of mechanotransduction^271^. To study this, several methods have been developed to deliver specific physical stimuli to organ-on-a-chip models, including fluid shear forces, compression, and stretch to achieve relevant maturation^272^.

**Fig. 3:**
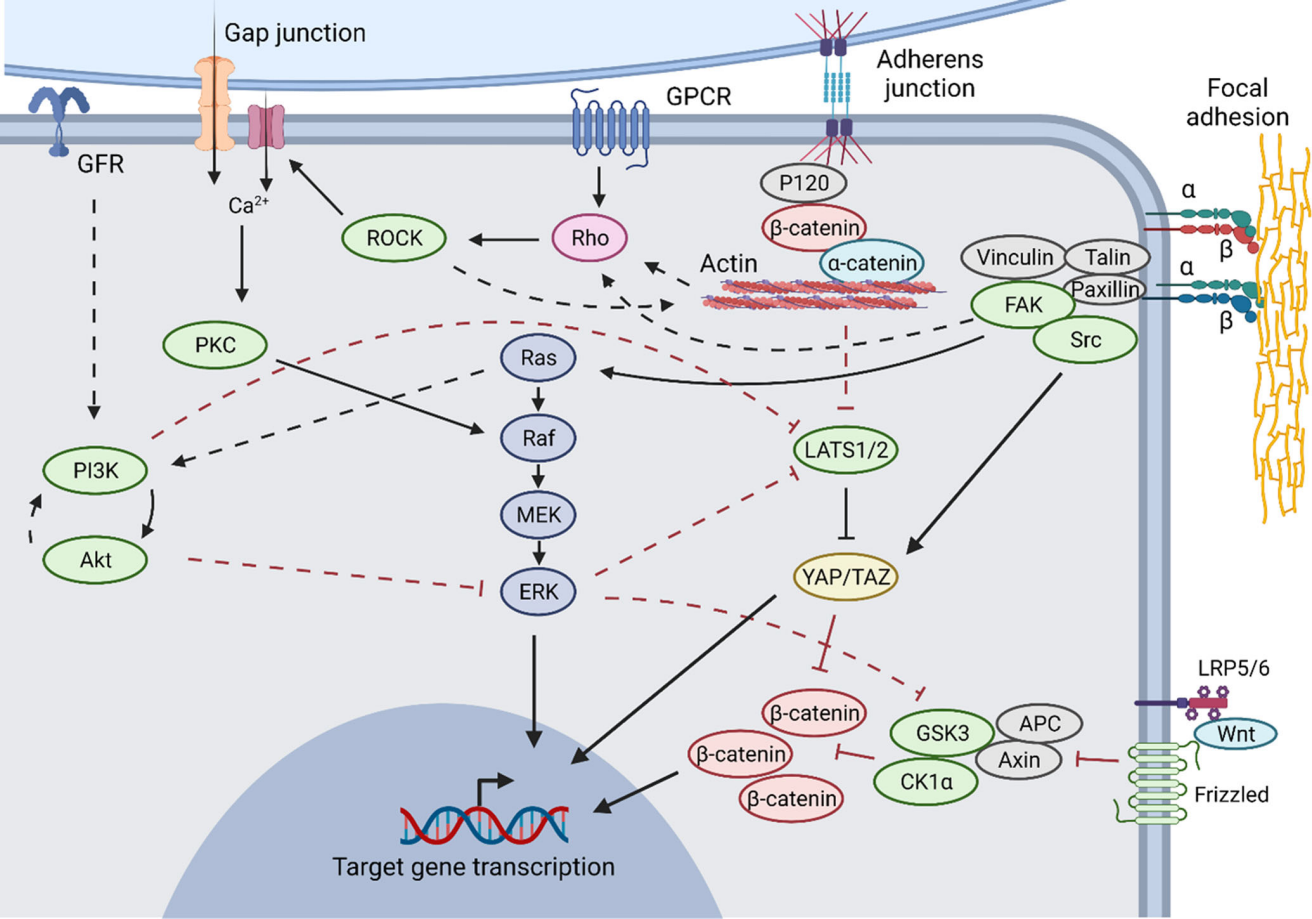
Convergences between physical signaling and common consolidated pathways. Black arrows represent activation while red hashes represent inhibition; solid lines represent putatively direct interactions while dashed lines represent indirect effects (intermediates not shown or unknown). Image created using BioRender.com.

Cell connections and environmental stiffness are well-known to be transduced through integrins and junctional complexes, in some cases dependent on MRTFs and YAP/TAZ signaling through the Hippo pathway^268,273^, while related Wnt/β-catenin signaling has been well-recognized in the development and maturation in many tissues^274–278^. Similarly, cadherins and ephrins are implicated in certain specific maturation events^279–281^, and may well be implicated in others as they are targeted for study in the future. Cadherins have specifically been shown to interact with the PI3K/Akt pathway^282–284^, suggesting that exogenous forces can modulate the transduction of other maturative signals that utilize these pathways (Fig. 3). Cell tension and other integrin signaling can be transduced through a focal adhesion kinase (FAK)-assembled complex (containing paxillin, Src, and talin among other proteins) affecting PI3K/Akt, MAPK, Src, and JNK pathways^273,285^, as well as integrin-linked kinase^286–288^, which can all exert maturative effects.

Nanotopography and ECM micropatterning have also proven to be a powerful method to exert control on cell fate and function in vitro in several applications^288–295^, in part by defining boundary conditions and so controlling the directionality of exogenous forces on a cell, as well as by regulating the formation of focal adhesions^296^. These concepts have been well-illustrated in the model of the intestinal crypt, which features diverse cell types in close proximity and a dynamic environment. Cell distribution and niche is highly organized, with specific gradients along the depth of the villus/crypt^297^ that likely correlate with a change in stiffness^298^. In the intestinal epithelium, integrin-linked kinase has been linked to cell motility, differentiation, and function^299^. Furthermore, an ephrin/Wnt/Notch signaling network has been implicated in both villus/crypt development and intestinal stem cell maintenance/differentiation^298^. Given the ubiquity of common mechanobiological signaling pathways in developing tissues^300^, it is likely that analogous processes can be found in other models. Physical signaling in vasculature exerts significant secretory and gradient effects on its environs (as discussed below). Signaling effectors of permeability are generally associated with calcium-dependent pathways (including PKC, Src family kinases, and RhoA)^301^, as well as the output metrics of polarity and alignment^302^. Shear forces have been also demonstrated to exert direct effects on function in models of the gastric epithelium^303,304^ and liver^305^.

ECM and physical signaling are perhaps most studied in context of fibrosis. Fibrosis occurs in some form across nearly all tissues, and generally represents loss of signaling and mechanical homeostasis followed by the re-establishment of a new equilibrium characterized by myofibroblastic maturation and responses by the other cells present in the tissue^306^. Although fibrosis has been directly studied in tissue models^307–310^, excess ECM deposition or pathological stiffening may need to be monitored in most if not all organoid models as they become more complex due to 3D form, perfusion, and co-culture, as such a positive feedback loop-response to a loss of homeostasis may mask, blunt, or subvert the physiology of models of either otherwise healthy function, or specific pathology.

In addition to cytoskeletal transduction of physical forces via cell-cell and cell-ECM connections, cells can respond to external forces using mechanosensitive ion channels including: Piezo1/2 channels, TREK/TRAAK K_2P_ channels, TMC, and TMEM63. These channels are present and active on a wide variety of tissues including myocardium, vasculature, hepatic sinusoids, bone, lung parenchyma, pancreas, cartilage, multiple glandular epithelia, nervous tissue, and renal tissue and urothelium^311–313^. Additionally, these channels may provide a significant contribution to shear stress mechanotransduction and baroreception^311–313^, of which definitive mechanisms have remained difficult to identify. With specific proven roles in development and maintenance of function (or pathology in their dysfunction), mechanosensitive ion channel^313,314^ and ROS activity^13,315,316^ likely exert a role on the maturation of cells and their composite tissues. However, the extent of their signaling activities, and local protein assemblies that mediate their effects, remain an active area of research.

In a tissue, many highly differentiated cells become connected in a functional syncytium via expressions of linking connexins. Connexin isoform expression patterns are tissue-specific, and so provide selective cell-cell interactions based on compatibility. Furthermore, connexin subtype gives control over passage of transjunctional small molecules based on size, shape, and charge; allowing regulated passage of second messengers and metabolites including but not limited to adenosine and its phosphates (cyclic) AMP, ADP, and ATP, and IP_3_^317^, as well as Ca^2+^, ROS, and NO^318^. Transit of these messengers (and thus oscillatory behavior, see above) between cells is geometry-dependent^319^, again reinforcing the importance of 3D biomimetic structure and co-culture (for connexons between different cell types), but also the convergence of cues towards holistic maturation (e.g., morphological maturation influences calcium flux between cells, which could have positive feedback toward further maturation, as discussed above). For example, physical pairing of an immature CM to a mature one in vitro can positively enhance the functional maturity of the immature one without further intervention^320^, suggesting the importance of cell connectivity in signaling.

### Molecular effectors of cell and tissue maturation

Although each tissue or cell type may respond more to certain key factors playing outsized roles in development, the realities of cell and tissue maturation (i.e., higher cell functionality) generally requires increased metabolic flux. As mitochondrial oxidation is both higher-yield in terms of ATP flux as well as more efficient in terms of energy extraction, most maturing tissues must activate mitochondrial biosynthesis; however, substrate preference can be more muted between tissues based on nutrient availability in vivo. Not surprisingly, the endocrine, paracrine, and physicochemical signals of the cell niche that induce these metabolic maturation processes are often conserved between tissues.

A postnatal switch in metabolism and the level of function occurs in many organs; postnatal shifts often coincide with weaning in animal models^138^, and may be associated on a tissue-specific basis with hormonal windows or profiles, nutrient shifts, changes to oxygen availability, and microbial exposure. This early generalization and later specification to an organism’s state may be reconciled on the level of natural selection, namely that organisms must adapt to specific challenges in their environment and specification; full functional commitment to a given nutritional or signaling niche before birth may disadvantage a newborn due to the resources needed to remodel tissues (e.g., based on available diet/nutrition, hormones in milk, etc.). This flexibility enables the high level of postnatal plasticity observed in many tissues, and is encouraging to tissue engineering efforts with the goal of replicating native tissue function; many embryonic windows and specific signaling events may not need to be met if the rich and specific postnatal niche is achieved in vitro to guide tissue-specific maturation. However, a ceiling on cell function, based on early stimuli, may exist. Similar to the phenomenon of epigenetically-enabled somatic cell memory as observed in iPSCs, oxygen availability^321^ and mechanical environment^293^, even at the pluripotent stage, can greatly influence functional performance.

Ultimately, despite tuning sensitivity toward many active maturation factors, those signals must be present to exert their influence, whether on an intracellular, autocrine, paracrine, endocrine, interstitial, or otherwise circulating basis. An example of metabolic regulation influencing receptivity to other maturation signals can be found with T3, a common effector of maturation. Thyroid hormone has numerous roles in tissue maturation and continued systemic metabolic regulation,^50,322^, and has been shown to be essential for the development of virtually all organs, including kidney, liver, neurons and BBB, other vasculature, and heart^322,323^, with evidence for very specific temporal and regional regulation, at least in brain^323^. T3 is transported at least in part via monocarboxylate transporters. Relatively little is known about the regulation of T3-active transporters, although a review of the available evidence suggests downregulated expression of transporters in both fasting and inflammation^324^. Alternatively, the nutritional state of the cell and the general endocrine/paracrine environment would certainly be relevant in T3 homeostasis. Even after the dynamics of T3 internalization, it must exert its effects in another dynamic system; the thyroid hormone receptor (THR) binds other nuclear receptors such as the retinoid X receptor^325^. However, in the absence of ligand, THR complexes with nuclear receptor co-repressors 1 and 2, among others. These co-repressors, once bound, oppose many transcriptional activities, including those of ERRs^326^; this suggests that THR activation may potentiate the pro-maturation effects of other pathways that are described in this text.

Glucocorticoid receptor (GR) biology has been noted as contributing to metrics of maturation in multiple tissues; several GR ligands have been identified to regulate downstream signaling by NF-κB, AP-1, STATs, and PPARs, among others^118^. This makes GR signaling an immediate subject of interest for possible synergistic, conflicting, or potentiating interactions with the other key pathways in maturation discussed above. Additionally, PPARα interacts with GRs; although GRα internalizes and activates genes implicated in lipid and protein metabolism, it also upregulates gluconeogenesis. Simultaneous PPARα activation will silence the transcription of genes regulated by glucocorticoid response elements^327^. However, at the same time, PPARα activation enhances active GRα binding and blockage of NF-κB and AP-1, and their associated transcriptional effects^327^, suggesting that the balance of signaling in terms of transcription factor activation is highly context-dependent and likely dependent on crosstalk from concurrent signaling processes. Continuous GRα activation is implicated in insulin insensitivity by decreasing insulin receptor substrate (IRS) expression^328^; this could be viewed as a positive reinforcing loop, as decreased insulin signaling will then result in even less downstream activity. PPAR binding of lipids^329^ is thought to stabilize protein-protein interactions and response element binding, although the low affinity may mean that lipid activation of PPAR is not significant under physiological conditions^329,330^.

The soluble and orphan (i.e. unknown endogenous ligand) ERR also activate PPARα/β response elements^16,41^. Although the ligands for ERRs are far from settled, ERRα activation by cholesterol has been established^331^, and other steroids or lipids remain potential ligands. The metabolic response to ERR activation^332^ is both dependent on receptor subtype and tissue in question, the latter ostensibly results from epigenetic influence or crosstalk with other active regulatory pathways. For example, in the hippocampus, ERRγ is heavily implicated in memory function through its enabling of greater glycolytic and glucose-based oxidative flux^333^, while in the heart ERRα/γ have been shown to downregulate glycolytic genes and simultaneously upregulating (FA) oxidative function^45^.

Finally, metabolic and maturation effects can often be attributed to one or more of many PKCs, with different dependences on Ca^2+^ or DAG (produced via phospholipase C activity) for activation, having downstream effects on motility and adhesion as well as FA storage and metabolism^334^. These widespread signals include both positive and negative interactions with insulin signaling through multiple control points, including the IRS, Akt, and other poorly elucidated targets. The net result of PKC signaling is, therefore, isoform- and tissue-dependent, and is likely afforded great flexibility due to the range of PKCs whose expression can be induced, which have been reviewed in detail elsewhere^335,336^. PKA and JAK/STAT signaling intersect with PKC^337^ and AMPK^338–340^ as well, especially at the level of transcription factor effectors.

### Metabolic signatures: substrate signaling fueling differentiated tissue function

During tissue differentiation and maturation, many cells reduce their endogenous biosynthesis of essential substrates and cofactors, relying instead on vascular supply of nutrients, hormones, and other signaling-active molecules from metabolic organs (e.g., the liver, pancreas, GI tract, etc.). Depending on the tissue in question, common carbon sources for energetic usage include glucose, lactate, lipids, ketones, or amino acids. However, substrate use, at steady-state, requires that bioenergetic and biosynthetic intermediates (NAD+ and NADP+, respectively) be regenerated to equilibrium with the cellular redox state, the former via either mitochondrial oxidation or anaerobic fermentation to lactate, and the latter via the completion of reductive anabolic steps. The preferential use of specific carbon sources, and the pathways implicated in this regulation, are highly dependent on the nature of the tissue function. Below we discuss tissue-specific examples of how cell types require a metabolic niche, and how the resulting metabolic niche of a tissue in turn affects signaling and function. Even signaling-inert molecules (i.e. non-ligands) can indirectly modulate relevant pathways by affecting key indices that influence homeostasis, such as pH, AMP/ATP ratio, NAD+/NADH ratio, redox status, etc. In the next sections of the review, we highlight both the central mechanisms underlying maturation, and several convergences with metabolism that can impact the functional phenotype of the tissue.

Similar to initial carbon sources, many intermediate metabolites or biological reductants are signaling-active; including intermediate carbon sources such as acetyl-CoA, succinyl-CoA, AMP/ATP, and glutamine^341^, likely among many others. Other metabolically-associated small molecules can influence the signaling state through simple equilibrium effects; increased acetyl-CoA concentrations in turn kinetically upregulate acyltransferase activity^190^. Similarly, changing the NAD+/NADH balance through redox state or nutrition can change sirtuin family deacylase activity^342^, as discussed above. More directly, many carbon sources can induce direct signaling cascades within targeted cell types. For example, ketone signaling can be extensive both via direct receptors, and via downstream response effects due to the implied metabolic state that influences ketone circulation and metabolism to begin with. Ketone signaling has been observed in neuronal, intestinal, vascular, adipose, and liver tissue^190^; acetoacetate activation of GPR43 indicates convergences with that receptor’s canonical lipid sensitivity^343^, and has also demonstrated crosstalk with spatial (Notch) signaling in intestine^344^. Another common ketone body, β-hydroxybutyrate (β-OHB), inhibits certain histone deacetylase activity and in doing so activates oxidative stress defenses to enable oxidative metabolism^345^, but conversely can also inhibit lipolysis in adipocytes via activation of GPR109A/HCAR2 (hydroxycarboxylic acid receptor 2, also known as niacin receptor 1)^190,346^. The antioxidant activity of β-OHB has been attributed to downregulation of NF-κB, ostensibly through IP_3_ activation and intracellular [Ca^2+^] increase. These signals will in theory interact with the Ca^2+^-sensitive processes identified above, and therefore be sensitive to cytosolic Ca^2+^ oscillations. Furthermore, GPR109A activity leads to both MAPK and PPARγ activation^347^, exerting additional control points on energetic and exogenous signal-linked pathways, as described previously. Given ketones’ roles as a metabolite of last resort during periods of low supply (e.g., fasting or starvation)^346^, it is likely that there are many ketone-dependent signaling processes organized per tissue that have not yet been fully characterized in context.

## Design strategies for complex processes: future directions for search processes, culture practices, and optimization strategies

To date, the study of in vitro tissue maturation has revealed few shortcuts to high functionality, suggesting that efficient and effective maturation is a multi-system process. As outlined in the early sections of this review, the convergence of tissue-specific cues, circulating nutrients and endocrine signals appear to coordinate progression across tissues to genetically-encoded checkpoints^348^. However, the available evidence suggests that functional maturation within a specific tissue is largely a holistic process, and that advancing one phenotypic metric is likely to advance others, both via shared transcriptional organization and through the development of cellular microdomains, and due to the self-reinforcing paradigm of cellular energetics. Advanced function requires specialized metabolism, which will only occur through metabolic insufficiency stress and ROS production, while increased energetic capacity will enable biosynthesis, structural complexity, and thus tuned and advanced function.

As a result of our limited understanding of the processes, pathways, and checkpoints underlying tissue maturation, it appears difficult to reach these functional milestones in vitro with current capabilities. Moreover, existing tissue engineering optimization strategies do not necessarily have the predictive power of novel approaches, either in interpolation or extrapolation. Below, three generalizable and actionable strategies for optimization of tissue maturity are outlined and discussed in depth. They include: replication of a tissue’s (bio)chemical niche, perfusable 3D co-culture, and design of experiments to account for biological complexity.

### Tissue-matching of the (bio)chemical environment

The (biochemical) balance of an in vitro environment is a longstanding and common method of biological model optimization, pioneered with the introduction of Ringer’s solution in the 1880s, continued with Earle’s salts in the 1930s, and providing a foundation for development of many common cell culture media in the 1950s-60s. Serum (e.g., fetal bovine, fetal porcine, or calf sera) has been traditionally used to provide both complex nutritional supplementation and growth factors, but carries several important limitations. Undefined culture media (e.g., containing serum at 5–10% v/v or greater) are not replicable and introduce batch effects into experiments^349^. Moreover, high medium serum content induces toxicity, whereas low serum does not provide enough essential nutrients at biomimetic levels^350^. Efforts to minimize the presence of effective but undefined growth factor content include the use of adult or supplemented calf sera, which are generally less effective for cell growth. In recent years, the rise of defined (*i.e*., containing isolated or purified xenogeneic components) and chemically-defined (*i.e*., containing only recombinant products beyond organic small molecules and inorganic components) supplements to ubiquitous basal media have overcome the use of serum-derived complete media for both replicability and translatability concerns. Several such supplements of note include the B-27 supplement, which was developed for neural culture but has been recognized for its efficiency in the maturation of diverse tissues^351–353^. Other supplements include insulin-transferrin-selenium, creatine-carnitine-taurine, serum replacements (such as KnockOut™), lipid-enriched and/or recombinant albumins, and chemically-defined lipid supplements, in a growing field of defined supplements^354^.

The near-ubiquitous use of media unspecialized to the ionic and nutritional niche of the cell or tissue in question has been recently examined^355^, warning that cell physiology may be compromised without carefully designed media for the situation at hand. In the context of tissue cultures, this suggests a benefit to the use of a conscious choice of basal media, and/or the use of properly perfused tissues with endothelium or co-cultures that benefit from vascular barrier function and ion-regulation (see below). Similarly, tissue function may be compromised in medium such that they cannot regulate their environment as expected in vivo. For example, astrocyte-mediated regulation of neural tissue extracellular fluid is extremely precise^356^. Changing the niche and scale, where the mass of cells cannot adequately regulate within said niche, would change the metabolic demands and thus the functionality of the cells.

Traditional culture media are also notorious for physiologically irrelevant levels of glucose in media (commonly 25 mmol L^−1^). For short-term cancer cell culture these levels may be largely inconsequential; however, the effects on putatively healthy cells with altered homeostasis may currently be underappreciated^355^, and could elicit magnified effects when used in delicate and interbalanced co-cultures. Included in these concerns are worries of oxidation and interactions with insulin-related signaling and effects, if present. Glycation can occur to functional effect on extracellular proteins within weeks^357^, which could in theory affect ECM, receptors, channels, or other extracellular-presenting proteins. Little work has been done on glycation or oxidative degradation of media, and most of it has been in the field of bioprocess; as a result, proliferation or recombinant protein production are the functional metrics of interest that show sensitivity to these processes and suggest that other emergent functions could as well^358^. Although advanced glycation end products can take time to appear, they may have rapid effects on cell function once present^359^; this is of particular interest not for traditional proliferative cultures, but for quiescent and highly functional tissue models.

The issue of glucose over-availability would not likely be addressed by simple replacement by a large quantity of a different carbon source, as toxic effects of other metabolic substrates may also exist; lipid supplementation can result in the relatively unelucidated phenomenon of lipotoxicity^41,360^, which generally manifests in ER, mitochondrial, or Golgi stress. The pathways of this toxic response can include JNK/Bcl2 family signaling as well as the well-known ER stress signaler PERK, leading to pro-apoptotic ATF4 activation^361^. Lipids also interact with cyclooxygenase and cytochrome P450 activities, both of which can elicit ROS production, or may result in unintended metabolite production, such as the ubiquitous FA, arachidonic acid, and its eicosanoid products^362^. Lipids may however carry additional benefits to complex co-cultures beyond specific functional effects; reports of lipid supplementation enhancing the secretion of angiogenic factors by mesenchymal stem cells^363^ suggests feedback loops when perfused co-cultures are combined with well-optimized culture media.

The use of carbon sources may need to be considered on a case-by-case basis, such as in personalized medicine screening. Ketone metabolism is unsustainable to certain CoA-transferase deficient patients in vivo^346^, and can be incompatible with certain metabolic or signaling states even in healthy tissue/patients. The use or avoidance of ketones may be important in eliciting a desired response, or in mitigating an unintended phenotype. These patterns may also hold true with lipid-related CoA-transferase or carboxylase deficiencies.

### Tissues benefit from co-culture: 3D structure, cell-mediated culture benefits, and gradient effects of perfusable vasculature

Tissues generally require a selection of cells to maintain utility; a complex mix of endothelial cells for perfusion, fibroblasts for interstitial maintenance, immune cells, and often specialized structural cells support the function of the nominally “functional cells” of a tissue. These cells often provide paracrine or mechanical support in situ, such as endothelial secretion of potent growth factors, and immune-directed tissue remodeling. In this way, the complementary functions of different cell types can drive each other to advanced function. There is compelling evidence that the inclusion of immune cells may be a powerful tool for tissue maturation due to their complex cytokine secretion profiles, and regenerative or pro-maturation programming paradigms^125,197,198,364,365^. However, there has not been widespread inclusion of immune cells in functional tissue models to date due to several factors, including difficulty in cell sourcing, complexity of phenotypes in tissues of interest, and lack of effective techniques to include immune cells in culture without inducing unintended response patterns. The growing recognition of the role of the immune system in healthy function and development, as well as pathogen defense, is likely to spur significant advances in our understanding of how both to harvest and implement immune cells in co-cultured engineered tissues.

The benefit of perfusable vasculature has been mentioned in several engineered tissue types above in the context of their respective secretomes and nutrient selection for cells outside the vascular lumen. Engineered vasculature may enable nutrient and oxygen supply in larger 3D cultures^104^, which through mechanical signaling alone can significantly influence cell fate. Subtle variations to nutrient and oxygen gradients can also have outsized effects^366,367^. Vasculature provides selective barrier function, and a massive secretome of growth factors, cytokines, and other small molecules. Moreover, endothelial function can be modulated by the perfusing chemical environment or the main functional cells of the tissue, such as CMs^368^, which will then feedback into emergent tissue function; endothelial permeability and smooth muscle tone can be modulated by histamine and platelet-activating factor, radicals such as nitric oxide or superoxide, and even nutrients such as ketones^347^. Oxygen gradients established in perfusable tissues have been previously discussed, and can differentially activate HIF1/2α signaling, which has been implicated in the differentiation, maturation, and maintenance of healthy tissue function in many cases^369^.

The inclusion of vasculature or considerations of mechanical replication of tissue features seemingly necessitates 3D culture, which is most typically aided by the use of hydrogels or scaffolds that are largely generated through synthetic means or using decellularized native ECM; polymeric scaffolds or gels will ultimately offer the highest batch replicability and lowest propensity for contamination, toxicity, or unelicited and divergent cell responses. However, 3D tissues also necessitate perfusion, as oxygen gradients to the interior of an engineered tissue quickly become unsustainable. Oxygen availability is not an issue limited to 3D culture; depending on media depth, specific metabolic rate, and cell density, static monolayer cultures can experience significant hyperoxia or hypoxia^34^. Paradoxically, either condition may induce ROS production^34,370^. While the source of ROS under hyperoxia is clearly driven by equilibria, hypoxia (a lack of oxygen) may also induce the production of superoxide, as ETC complexes are forced to remain in a reduced (i.e., electron-rich) state^371,372^; increased ROS may either cause a toxic response in the face of insufficient mitigation, or require a constant and significant metabolic expenditure in the form of biosynthetic reductant (NADPH) to maintain antioxidant defenses depending on the cells or tissue in question. Mature tissues, which rely heavily on oxidative flux to maintain function, will likely have developed significant cellular infrastructure that is then allocated to maintaining ROS within acceptable levels; ROS are not uniformly harmful to cells and are key in homeostatic signaling in healthy tissue^373,374^. Therefore, the appropriate provision of substrates, cofactors, and antioxidants in the media may need to be optimized for the tissue model in question as insufficient levels of ROS may equally inhibit nominal cell function or maturation processes^375^.

### Experimental design to reflect and harness biological complexity

In recent years, the ability to craft in vitro tissues of functional relevance has increased rapidly, and commensurately with advances in cell and developmental physiology, microfluidic and organoid culture, and biomaterial development. However, recapitulating the dynamic nature and inherent complexity of a living functional system is extraordinarily difficult, and reaching higher levels of mature function has to date required progressively greater investment of time and resources in cell biology and biomaterials science. Moreover, the analysis and benchmarking of further-matured engineered tissues requires greater precision, resolution, and standardization in methods, all of which require greater investment and training in relevant equipment and techniques. As these burdens become greater, the ability to screen cultures by traditional means may present a serious barrier to continued innovation in tissue engineering, or ever fully achieving high-throughput tissue engineering in a non-industrial setting. Furthermore, experimental infeasibility may impose limitations on innovation where progress to tissue maturity may represent more of a sigmoid of function against time, where the upper bound is not adult in vivo function but rather limited or idiosyncratic function. However, this putative limitation may be more likely to be realized under widespread continuation of the prevailing traditional prototypical design strategies (one-at-a-time, single version, or design-of-experiment processes) of tissue engineering, that struggle to model all of the dynamic and interrelated processes of a living cell or tissue (i.e., a complex system, characterized by nonlinear responses, interaction between factors or outputs, or hidden variables). The physiological complexity of any functional tissue likely precludes its engineering by traditional design methodologies, e.g., without the use of significant screening phases for relative cell composition and source, the inclusion of ECM and other biomaterials, perfusion and oxygenation, and (biochemical) niche. In these screens, simple morphological, expressional, and transcriptional metrics might generally be replaced in scoring regimens by emergent functional metrics specific to the tissue in question, reflecting the final goal of mature tissue. When multiple such metrics are available, decisions must be made concerning how to construct a complexed desirability function, and whether iterative decision making is valuable during an optimization.

**Fig. 4:**
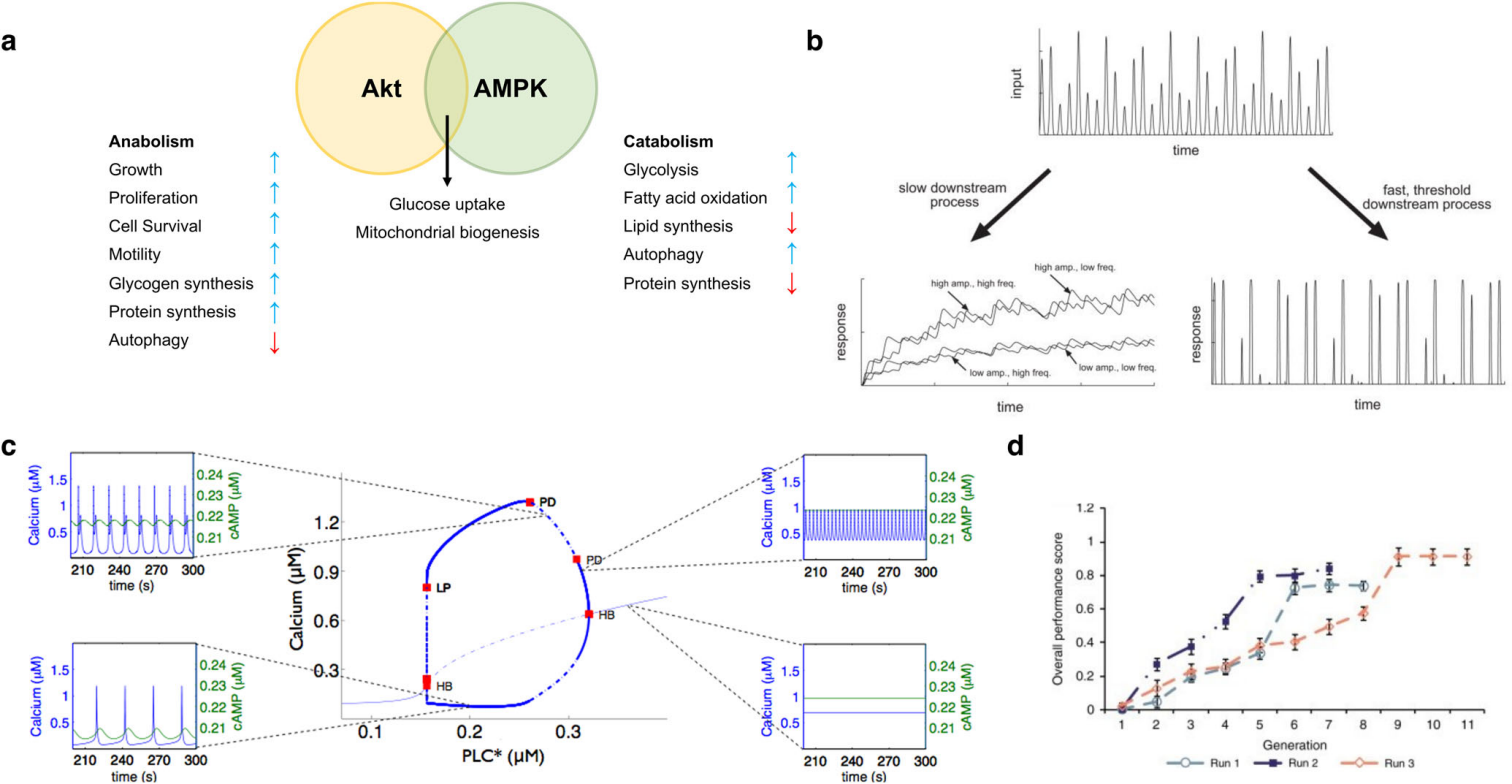
Interdependency of consolidating signal transduction pathways, and oscillatory signaling processes and second messengers coordinating tissue function, in introducing complexity when setting optimization metrics, prescribed signaling targets, or scoring paradigms. **a** Opposing and aligned roles of Akt and AMPK in cell processes. **b** Oscillatory inputs (e.g., [Ca^2+^] or [cAMP]; top) can be decoded on an amplitude-dependent (left) or frequency-dependent (right) basis based on receiver process kinetics being slow or fast, respectively, lending further mutability to the physiological manifestation of a set of interdependent and multi-step signaling pathways^376^. Reproduced with the express permission of the publisher and authors. **c** Simulated interactions between calcium and cAMP levels in a model of mutual enzymatic activation, based on active phospholipase Cβ (PLCβ) concentration and a set adenylate cyclase concentration of 0.17 µM^248^; demonstrating interdependency of second messenger kinetics on their ultimate manifestation as an output response. Reproduced under a CC-BY license with express permission of the authors. **d** Sample visualization of an iterative High Dimensional, Differential Evolutionary (HD-DE) algorithm with successive generations outperforming each other on a normalized objective metric, with biological variability represented by temporary differences in performance between independent iterative runs that ultimately converge at highly-performing solution scores; this algorithm was used to optimize hematopoietic stem cell expansion medium efficacy^388^. Reproduced under a CC-BY license with express permission of the authors.

Predicting or realizing a specific defined signaling state related to maturation may be exceedingly difficult, due to crosstalk between signaling pathways, second messengers, and the mutability of the related physiological decoding processes or responses (Fig. 4), such as the previously discussed balance between the master signal consolidators Akt and AMPK (Fig. 4a). For example, the response of a cell or tissue to a complex signaling input can be reliant upon the receptor that decodes it and the kinetics of the response process itself (Fig. 4b)^376^, while composite signals can be highly interdependent (Fig. 4c)^248^. Depending on the kinetics of the response process, this means that the output level of the response can be constitutively elevated or depressed (e.g., cumulative amplitudes of signals giving rise to the slow downstream process example in Fig. 4c, which might be elicited in cell fate, expressional, secretory, or migratory functions that occur on timescales that are many times longer than a discrete signaling event), or subject to different output frequencies or magnitudes (e.g., the fast, thresholded downstram process). A putative example of the difference between these modalities of response might be found in the myocardial force response to filling. Here, fast [Ca^2+^]-induced beat-to-beat variation in mature myocardium^377–380^, has been shown to be mechanistically distinct from slow (e.g., minute-to-minute scale) adaptations to preload^381–383^; the ability to mount these responses is intrinsically tied to the healthy function of mature myocardium. In the absence of a well-validated signal definitively correlated to maturation, even establishing the correlation of any one measurable signal (e.g., a (de)phosphorylated signaling enzyme, a second messenger concentration, transcript or protein expression, etc.) within relevant signaling cascades may become opaque. The use of prescribed or otherwise actionable signaling states, especially as maturation levels increase and in theory become ever more highly- regulated and sensitive to disruption, might lose utility or even become misleading without the use of neural networks or other AI methods necessary to dissect complexity, in combination with large relevant datasets. For this reason, metrics of cell or tissue physiological function provide a relatively holistic, robust, and correlative insight into maturation state and progress, and are likely to be of more use in optimization workflows of maturation in tissue engineering.

Traditional optimizations focusing on one factor at a time can fail to model or account for important multi-factor processes that occur in complex systems. However, tissue engineering and biology in general are notorious for uncontrollable or unidentified factors, batch effects, and higher-order interactions that get missed using simple experimental designs or optimizations. Factorial designs (e.g., Design-of-Experiments methodologies) have been implemented to a degree in tissue engineering studies, but are often constrained to very high-level problems and have varying degrees of success at optimization, due to throughput limitations in testing experimental runs which scale exponentially with the factorial complexity of the system in question; these optimization experiments also tend to lose resolution quickly with experimental variability. However, other more-“established” (i.e., greater industrial footprint, formalized practice, and longer history as a recognized field) engineering fields make varying use of many tools to account for complex systems, including fuzzy decision making (i.e., decision making in an environment where the goals or constraints are imprecisely or otherwise vaguely designed or understood; notably a decision problem can be fuzzy even in the presence of highly quantitative or absolute data inputs), the use of objective metrics to encapsulate classes of function, iterative design-benchmarking-evaluation cycles, and holistic multi-criteria optimization (e.g., a weighted composite desirability function as the objective function^384^, or the use of Pareto fronts to identify acceptable trade-offs between different desirable outcomes^385–387^).

The challenge of efficiently performing high factorial optimizations, as described above, lies in the presence of nonlinear factor main effects, hidden interactions between factors, and the need for many experiments to statistically deconvolute a predictive model. An option for such problems in tissue engineering projects may lie in the adoption of biologically-inspired natural selection or directed evolution algorithms as embraced for chemical, molecular biological, and bioinformatic workflows, or a combination thereof (Fig. 4d)^388^. These processes hold in common that they do not seek to model the precise relationships between all inputs and outputs, but instead assess output functionality. In complex system, the harnessing of complex factor interactions and the use of limited stochasticity can enable a project’s benefit from iterative successes while navigating away through the solution space from failures. Separate from biological complexity, the realization of highly functional tissue-engineered constructs may involve significant handling of fuzzy problems, which are well-documented in other engineering applications^385,389^. Although the two concepts are often correlated or conflated, fuzziness in the case of tissue engineering would denote imprecision in the necessary and robust functional behavior toward maturation of a tissue model for downstream application, and in practice represents consensus on an acceptable end product without agreement on how to achieve it, and may conflict with the use of even multiple low-level quantitative physiological metrics with unclear connection(s) to emergent functional phenomena (e.g., the use of multiple cytoskeletal transcript counts as opposed to a contractile endpoint). To date, given the wide range of physiological metrics, both functional and not, fuzziness tends to increase when correlational metrics are more heavily relied-upon (e.g., expressional and transcriptional markers, morphology, etc.); whereas absolute metrics e.g., the peak contractile stress of a myocardial construct as a percentage of adult ex vivo sample stresses) tend to reduce the need for fuzzy decision making, and absolute thresholds can be constructed (e.g., being able to recapitulate >50% of adult contractile stress would be acceptable to stakeholders to leave the developmental cycle and begin using a model for downstream patient-facing applications). In either case, multiple parameters of a system, both functional and logistical, are constantly being weighed against each other during experimental design, either explicitly or implicitly (Table 8).

**Table 8:** Constraints in experimental design or system optimization in cell culture and tissue engineering.

Constraint class	Examples of metrics to assess in optimization scheme
Cost	Culture medium
	Culture device
	Cell source
Reliability	Batch variability
	Cell line variability
Predictivity	Sensitivity to differentiate disease
	Sensitivity to differentiate toxicity
User	Time requirement to use
	Skill requirement to use
Time	Lead time to prepare experiment
	Culture time to measurement
Biological function of cell/tissue	Catalysis
	Secretion
	Electrophysiology
	Barrier function
	Perfusion
	Contractility
	Morphology
	Transcription
	Protein expression

An alternate way to produce a Pareto-optimal solution toward two or more correlated but not identical outputs is using multiple decision makers^386^, which will closely approximate the output, if not methodology, of a composite desirability function. However, the use of decision makers still intrinsically includes an element of fuzzy decision making, and may provide a valuable workflow incorporating highly specialized advisors (e.g., a microfluidics engineer and an internal medicine physician) balancing their respective priorities in the realization of a functional tissue model. The use of one or more decision makers can be further formalized in interactive and iterative multi-objective optimization workflows^387^ that alternate phases of decision making with optimization. This in turn allows for response surface exploration during the developmental process; the non-orthogonality of factors and potential needs for variate re-weighting, assay re-optimization for a changing tissue, or sensitivity or variance testing are potential concerns for tissue engineering process optimizations due to biological complexity. Ultimately, certain objectives may come in conflict with each other, or the relative priority of one objective over another is unclear. In these cases, a Pareto-optimal solution that is acceptable to all stakeholders can be established through defined means^385,389^. For example, the set of possible solutions can be pruned or iteratively sorted by re-weighting existing parameters, introducing new factors of interest to increase dimensionality in a more focused solution space, or assessing logistical capacity or technical thresholds of interest.

Regardless of the level of complexity and fuzziness in a tissue engineering problem, the objective functions used for optimization will therefore likely be most effective when they represent a reference to or recapitulation of emergent physiology or relevant clinical metrics (especially in comparison to reference ex vivo metrics where available and appropriate). These metrics may include, depending on the tissue in question, protein or hormone excretion, ion reuptake, metabolic processing, barrier function and selectivity, electrophysiology waveforms and metrics, or contractility. Aside from best experimental practices in using functional metrics for designing engineered tissues, the best practices in optimization statistics and decision making under complexity, and especially in the case of multiple competing outputs, therefore recommend stepwise, stakeholder-driven and -corrected trajectories^385,386,389^. This can include re-weighting of composite desirability metrics, switching objective metrics, and insertion or termination of seeds/paths, depending on the model of decision making used. As the difficulty in advancing tissues in functional maturity increases (a trending theme made clear in the early sections of this review), the incentive to perform highly economical and well-planned experiments will correspondingly increase. As with other fields of engineering, the introduction of formal development methods that allow for multivariate optimization and the harnessing of complexity will likely produce significant advances to tissue engineering and its downstream applications.

## Conclusions

The mechanisms underlying functional tissue maturation in vivo require further elucidation, and currently this lack of actionable understanding may limit the development of highly functional engineered tissues for use in drug testing, basic science, or even graft applications. However, the use of systems biology approaches both in vivo and in vitro have yielded notable mechanistic understanding and innovation in tissue engineering studies. As a high level of function is invariably energy-intensive, most highly functional tissues (that are of immediate interest for engineering) are oxidative, and therefore rely on common trends of strict morphological regulation, high mitochondrial biogenesis, maintenance, spatial organization, and intracellular energetic shuttling to active organelles or regions. We hypothesize that by incorporating considerations of preferred substrates, hormones, cofactors, and potentially synthetic signaling-active molecules, a robust and self-perpetuating homeostasis can be established to push engineered tissue to higher levels of functional maturity. This goal may be accelerated by, or may require the use of, 3D organoids, perfusable vasculature, and/or co-culture systems to recapitulate a base level of function.

Finally, the inherent complexity and difficulty in articulating quantitative goals for adoption of a tissue-engineered model for downstream applications may pose a limit to the relatively simple experimental designs, design frameworks, and object-based decision making commonly used in tissue engineering today. The aims of the tissue engineering field instead likely necessitate the adoption of optimization and decision making frameworks common in other engineering fields. We have introduced potential study and decision paradigms and frameworks that may be of value in attempting to produce highly mature tissue models.

The ultimate goal of tissue engineering will be to mirror healthy and pathological function with near-indistinguishability from in vivo physiology. However, these ambitious goals do not need to be fully achieved before engineered tissue function becomes clinically relevant. The widespread introduction of iPSC-derived cells to replace animal models for preclinical drug screening aims to reduce both false positives and false negatives for both efficacy and toxicity endpoints. Moreover, the specifics of many human pathologies will never be fully recapitulated in animal models. By appreciating and accounting for the complexity inherent in living tissues, the field can advance models that interpret environmental stimuli and establish for themselves a homeostasis that recapitulates full mature function.
